# Interactions of Polychlorinated Biphenyls and Their Metabolites with the Brain and Liver Transcriptome of Female Mice

**DOI:** 10.1021/acschemneuro.4c00367

**Published:** 2024-10-11

**Authors:** Amanda J. Bullert, Hui Wang, Anthony E. Valenzuela, Kari Neier, Rebecca J. Wilson, Jessie R. Badley, Janine M. LaSalle, Xin Hu, Pamela J. Lein, Hans-Joachim Lehmler

**Affiliations:** † Department of Occupational and Environmental Health, University of Iowa, Iowa City, Iowa 52242, United States; ‡ Interdisciplinary Graduate Program in Neuroscience, University of Iowa, Iowa City, Iowa 52242, United States; § Department of Molecular Biosciences, University of California, Davis, California 95616, United States; ∥ Department of Medical Microbiology and Immunology, University of California, Davis, California 95616, United States; ⊥ Gangarosa Department of Environmental Health, Emory University, Atlanta, Georgia 30329, United States; # Interdisciplinary Graduate Program in Human Toxicology, University of Iowa, Iowa City, Iowa 52242, United States

**Keywords:** polychlorinated biphenyls, multiomics, RNA sequencing, metabolomics, network analysis, neurotoxicity

## Abstract

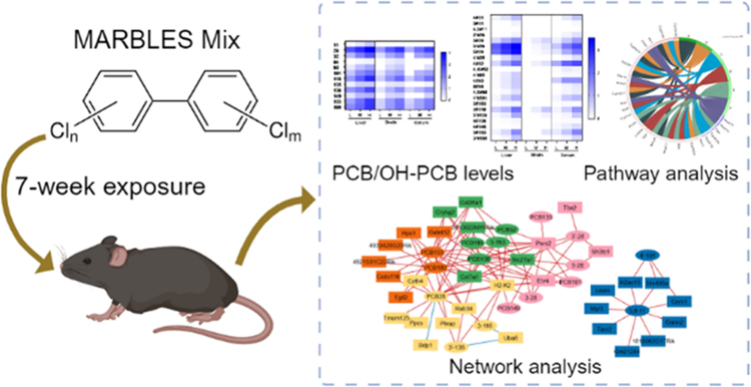

Exposure to polychlorinated biphenyls (PCBs) is linked to neurotoxic effects. This study aims to close knowledge gaps regarding the specific modes of action of PCBs in female C57BL/6J mice (>6 weeks) orally exposed for 7 weeks to a human-relevant PCB mixture (MARBLES mix) at 0, 0.1, 1, and 6 mg/kg body weight/day. PCB and hydroxylated PCB (OH-PCBs) levels were quantified in the brain, liver, and serum; RNA sequencing was performed in the striatum, prefrontal cortex, and liver, and metabolomic analyses were performed in the striatum. Profiles of PCBs but not their hydroxylated metabolites were similar in all tissues. In the prefrontal cortex, PCB exposure activated the oxidative phosphorylation respiration pathways, while suppressing the axon guidance pathway. PCB exposure significantly changed the expression of genes associated with neurodevelopmental and neurodegenerative diseases in the striatum, impacting pathways like growth hormone synthesis and dendrite development. PCBs did not affect the striatal metabolome. In contrast to the liver, which showed activation of metabolic processes following PCB exposure and the induction of cytochrome P450 enzymes, the expression of xenobiotic processing genes was not altered by PCB exposure in either brain region. Network analysis revealed complex interactions between individual PCBs (e.g., PCB28 [2,4,4′-trichlorobiphenyl]) and their hydroxylated metabolites and specific differentially expressed genes (DEGs), underscoring the need to characterize the association between specific PCBs and DEGs. These findings enhance the understanding of PCB neurotoxic mechanisms and their potential implications for human health.

## Introduction

Polychlorinated biphenyls (PCBs) are a class of structurally diverse industrial chemicals characterized by a biphenyl structure with one to ten chlorine substitutes. PCBs were sold in the United States under the trade name “Aroclor” as complex mixtures containing more than 100 individual PCB congeners.^1^ They possess unique properties, such as high-temperature resistance and chemical stability, making them suitable for various applications, including electrical, heat transfer, and hydraulic equipment.^2,3^ They are also present in plastics, rubber products, pigments, dyes, and carbonless copy paper. The United States banned the production of PCBs in the late 1970s due to their health risks and persistence in the environment. However, PCBs continue to be produced inadvertently.^4^ PCB congeners released from sites with legacy contamination and inadvertent PCBs continue to be a significant public health concern because of their continued presence in the environment. Human exposure to PCBs primarily occurs through contaminated foods, air, and dermal contact.^1,2^ Once absorbed, PCBs are metabolized by cytochrome P450 enzymes to OH-PCBs. PCBs and OH-PCBs exhibit toxic effects and are linked, for example, to cancer and disruptions in endocrine and neurologic functions.^2,3,5^

Mixtures of PCB congeners are present in the human brain,^6^ and laboratory and epidemiological studies consistently report associations between PCB exposure and impairments in learning, memory, and behavioral outcomes in children.^7,8^ PCB congeners that likely contribute to adverse neurodevelopmental outcomes were identified as part of the Markers of Autism Risk in Babies–Learning Early Signs (MARBLES) study,^9,10^ a study of women with increased risk of having a child with a neurodevelopmental disorder.^11^ A series of preclinical studies characterized the developmental neurotoxicity of the MARBLES mix, a synthetic PCB mixture approximating the PCB profile found in the serum of the MARBLES population. Wild-type mice or transgenic mice expressing either a human gain-of-function mutation in ryanodine receptor 1, a human CGG premutation repeat expansion in the fragile X mental retardation gene 1, or both mutations (DM mice) were exposed throughout gestation and lactation to the MARBLES mix at 0.1, 1, and 6 mg/kg/d via the maternal diet.^9,12−14^ These doses were selected based on earlier studies demonstrating that exposure to 6 mg/kg/d via the maternal diet resulted in PCB brain levels in weanling rats comparable to human PCB levels in the brain^15^ and that the lower PCB doses elicit human-relevant behavioral deficits in a mouse model.^9^

Behavioral assessments performed as part of these studies demonstrated reduced ultrasonic vocalizations at postnatal day 7 (P7) in wild-type but not transgenic mice exposed developmentally to these three doses of the MARBLES mix, suggesting disrupted early social communication skills.^9^ Developmental exposure to the low dose of the MARBLES mix significantly increased self-grooming at P25-P30 and decreased sociability in male wild-type mice at P27-P32. PCB exposure did not affect these behaviors in the female wild-type mice, regardless of the dose. Golgi staining was used to assess the effect of developmental exposures to the MARBLES mix on the dendritic arborization of pyramidal neurons in the hippocampus and cortex of mice exposed to the MARBLES mix via the maternal diet.^12^ A main effect of the MARBLES mix was identified using a multilevel linear mixed-effects model, driven by increased dendritic arborization of cortical neurons in the 1 mg/kg PCB dose group. The MARBLES mix also increased the dendritic arborization of cortical neurons of wild-type males in the 6 mg/kg PCB dose group. The MARBLES mix did not affect the dendritic arborization of hippocampal neurons in male or female wild-type mice. Developmental exposure to different doses of the MARBLES mix via the maternal diet also affected cytokine levels in mice with a mean age of P29.^13^ Briefly, serum but not hippocampal levels of T cell cytokines and innate inflammatory cytokines and chemokines increased with increasing PCB dose in wild-type and transgenic mice.

The available evidence demonstrates that exposure of mice to the MARBLES mix causes developmental neurotoxicity in mice exposed to this mixture via the maternal diet. However, it is largely unknown how exposure to PCB beginning in adolescence affects the mouse brain.^16^ The present study leveraged samples from female wild-type mice that were exposed beginning at approximately 6 weeks of age to the MARBLES mix as part of the developmental neurotoxicity studies^9,12−14^ but did not get pregnant. The goal was to explore how exposure to an environmentally relevant PCB mixture affects the striatum and prefrontal cortex, brain regions implicated in PCB neurotoxicity,^8,16^ using congener-specific PCB and OH-PCB analyses and transcriptomic and metabolomic approaches. Our study detected PCBs and OH-PCBs in the brain, identified alterations in gene expression pathways, and identified PCB and OH-PCB congeners correlated with changes in the expression of specific DEGs in the striatum and prefrontal cortex using network analysis. OH-PCB profiles in the liver and serum differed from those in the brain. Moreover, transcriptomic analyses in the liver indicate significant changes in metabolic regulations, for example, of arachidonic acid metabolic pathways, that may affect brain health via the liver-brain axis. These findings lay the groundwork for future mechanistic studies that characterize the role of specific PCBs or their metabolites on neurotoxic outcomes in rodents and, ultimately, humans exposed to complex PCB mixtures.

## Results and Discussion

### PCB Tissue Profiles and Levels

**Figure 1:**
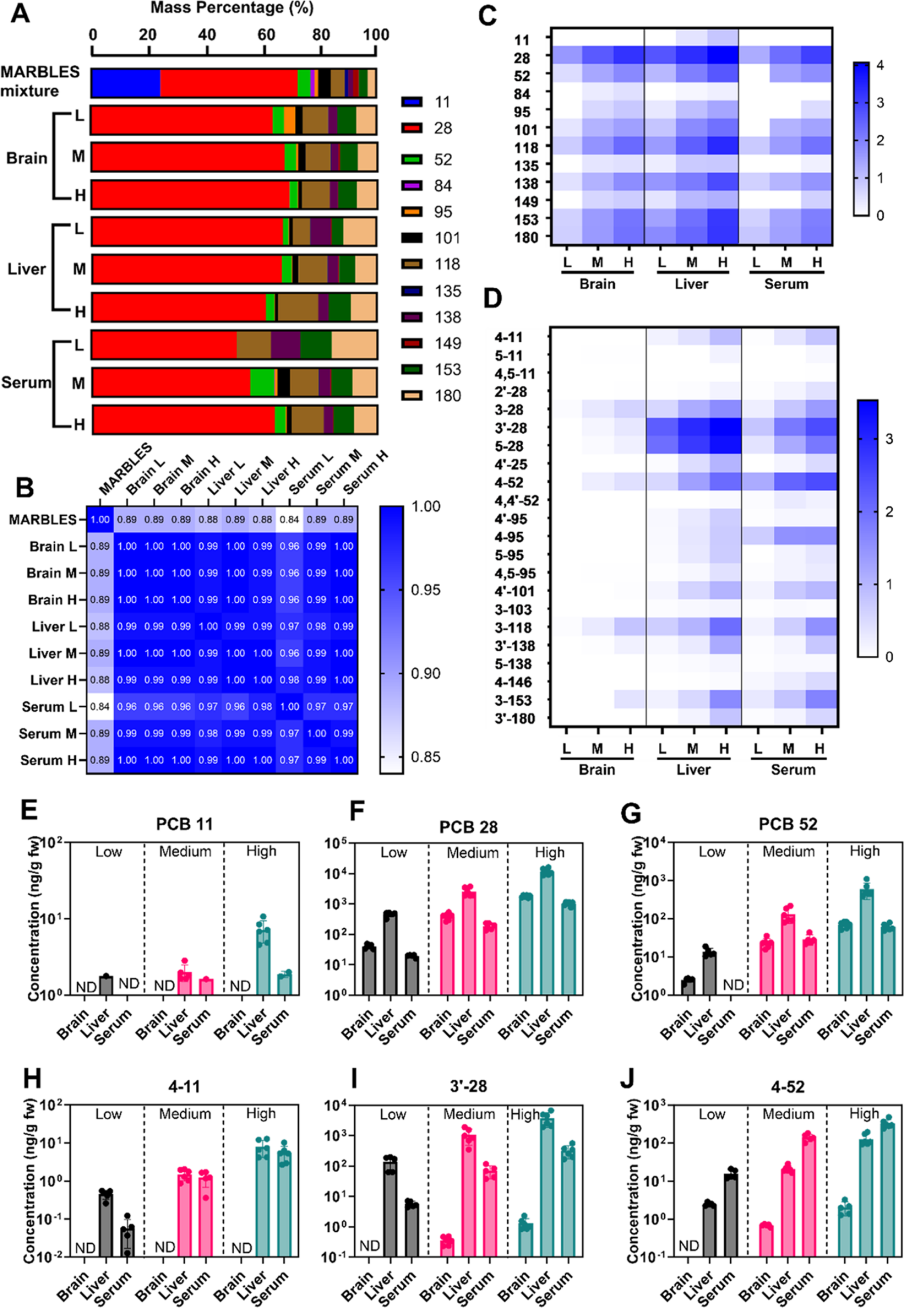
PCB congener profile of the MARBLES mix differs from the profiles of the PCB tissue residues, as illustrated using (A) a stacked bar diagram and (B) a heatmap-like comparison of similarity coefficient cos θ between PCB congener profiles in different tissues and the MARBLES mix. PCB congener profiles are expressed as the mass percentage %. The tissue levels of the MARBLES PCB congeners and the corresponding OH-PCB metabolites depend on the dose and tissue. Heatmap-like illustrations of (C) PCB and (D) OH-PCB metabolite levels (ng/g, expressed on a log scale) from all exposure groups in the brain, liver, and serum typically show a dose-dependent increase in PCB and OH-PCB metabolite levels. Levels of representative PCB and OH-PCB congeners, including (E) PCB11, (F) PCB28, (G) PCB52, (H) 4–11, (I) 3′–28, and (J) 4–52. All values in the heatmap are log-transformed, and values in the bar graph are mean ± SD of the fresh-weight adjusted levels, with each value represented with an individual dot. Differences in PCB and OH-PCB levels by dose and tissue were assessed using 2-way ANOVA, followed by Tukey post hoc analysis, with *p* < 0.05 considered significantly different, and are summarized in Tables S2 and S3. H, high dose; L, low dose; M, medium dose; ND, not detected.

Young adult female mice were orally exposed to different doses of the MARBLES mix for 7 weeks. PCBs and their metabolites were then measured by gas chromatography with tandem mass spectrometry (GC-MS/MS) in tissues and serum 24 h after the last PCB exposure (Figure 1). The mass profiles of 12 PCB congeners in tissues were similar across PCB doses but differed from the profile of the MARBLES mix, with cos θ ranging from 0.84 to 0.89 (cos θ = 1 indicates that profiles are strongly identical) (Figure 1A,B). These differences in the PCB congener profiles are due to the low mass percentage of PCB11 in the tissue residues. PCB levels typically increased dose-dependently in all tissues investigated (Tables S1 and S2), and the average levels in different tissue types followed the rank order liver > serum ∼ brain (Figure 1C). A dose-dependent increase in total PCB levels was also reported for brain tissue levels in postnatal day (PND) 32 pups exposed during gestation and lactation to the MARBLES mix via the maternal diet.^9^

PCB11 is a potentially neurotoxic constituent of the MARBLES mix based on laboratory studies demonstrating that it promotes dendritic arborization and axonal outgrowth in primary rat neurons.^17^ PCB11 accounts for 24% of the total PCB in the MARBLES mix, but PCB11 was not detected in the brain from any exposure group (Figure 1E). Similarly, PCB11 in the brain of PND32 pups exposed during gestation and lactation to the MARBLES mix via the maternal diet had a low detection frequency, with PCB11 levels ranging from not detected to <1 ng/g tissue wet weight.^9^ PCB11 was not detected in the serum of the low-dose group and was detected in 20 and 33% of the serum samples from the medium and high-dose groups, respectively (Figure 1E). In the liver, PCB11 only contributed to <0.1% of the total PCB. These results are consistent with the rapid biotransformation of PCB11 in cells in culture^18^ and disposition studies in rodents following oral and inhalation exposure to PCB11.^19,20^

PCB28, a lower chlorinated PCB congener implicated in adverse cognitive effects in older Chinese women (ages 61–90),^21^ was strongly retained in all tissues, including the brain, and accounted for more than 50% of the total PCB in these tissues detected (Figure 1A). Similarly, a disposition study in rats reported a 9- to 16-fold higher level of hepatic accumulation of PCB28 compared to PCB101 after intraperitoneal injection.^22^ The PCB28 half-life has not been reported in animal models. However, the half-life of PCB28 in humans was estimated to be 2.18 (95% confidence interval: 1.91–2.54) years^23^ and 4.32 years (95% confidence interval: 2.95–8.12 years)^24^ in PCB-exposed populations.

The second most abundant PCB congener detected in the tissues was PCB118, followed by two higher-chlorinated (≥5 chlorine atoms) biphenyls, PCB180 and PCB153 (Figure 1A,C). PCB118 is an aryl hydrocarbon receptor (AhR) agonist^25^ and causes thyroid cell dysfunction in cells in culture.^26^ Although altered thyroid hormone homeostasis is a potential mechanism for PCB-mediated developmental neurotoxicity,^27^ developmental exposure of mice to the MARBLES mix did not affect TH levels,^9^ and the PCB congener in the MARBLES mix or the MARBLES mix were neither agonistic nor antagonistic at the thyroid hormone receptor.^10^ The levels of PCB118 in the liver were almost 1 order of magnitude higher than in the brain and serum. PCB95, a congener considered to be neurotoxic by altering Ca^2+^ signaling via sensitization of ryanodine receptor (RyR) activity,^8^ was detected at a comparatively low level in all tissues (Figure 1C).

### OH-PCB Tissue Profiles and Levels

OH-PCBs have been detected at low levels in the rodent, Japanese Macaque, and human brain.^6,28,29^ In the present study, eight OH-PCBs were detected in the brain (Figure 1D, Table 1). In contrast, twenty-one OH-PCBs were detected in the serum and 15 in the liver. OH-PCB levels in the brain were typically lower compared to serum and liver (Tables S1 and S3). The lower OH-PCB levels in the brain may be due to the blood-brain barrier,^30^ which prevents the penetration of OH-PCBs into the brain, or differences in the tissue composition that, as we have reported for PCBs, affect the partitioning between blood and brain tissue.^31^ Based on a comparison of the average OH-PCB levels, a PCB 118 metabolite, 3–118, had the highest levels in the brain, followed by 3–28 > 4–52 > 3–153 > 3′–28 > 5–28 > 3′–138 > 4′–101 in the 6 mg/kg/d exposure group. The detection of PCB28 metabolites is consistent with the observation that OH-PCB28 metabolites are formed by cytochrome P450 enzymes in drosophila^32^ and their presence in human plasma.^24^ 4–11, a human-relevant PCB11 metabolite^33^ that increased axonal and dendritic growth in neurons in culture,^34^ was not detected in the brain. The presence of 4–52, a human-relevant PCB52 metabolite,^35^ in the brain for the higher exposure groups is noteworthy because this OH-PCB congener and its sulfate metabolite are toxic to neural and astrocyte cell culture models.^36,37^ Although PCB180 and PCB101 levels were relatively high in the brain, no hydroxylated PCB180 and PCB101 metabolites were detected in any exposure group.

**Table 1:** Profile of OH-PCB in the Tissues (A) Using Mass Percentage (%) and the Similarity Coefficient (cos θ) of the OH-PCB Profile (Panel B) across Different Tissues from Mice Exposed to 0.1 (L), 1 (M), and 6 (H) mg/kg Body Weight/Day of the MARBLES Mix^a^

**panel A: OH-PCB profile in mass percentage (%)**									
	brain			liver			serum		
OH-PCB congener	L	M	H	L	M	H	L	M	H
4–11				0.20	0.09	0.12	0.18	0.43	0.60
5–11	0.25	0.04	0.01			0.01			0.03
4,5–11						0.01	0.01		0.01
2′–28		0.09	0.07	0.01	0.01	0.01	0.21	0.15	0.17
3–28	64	34	20	1.1	0.91	0.75	2.2	2.2	2.4
3′–28		11	8.6	60	63	58	18	25	35
5–28		3.8	4.6	35	33	36	5.0	5.5	8.9
4′–25				0.05	0.14	0.15		0.24	0.33
4–52		18	11	1.1	1.3	2.0	50	50	36
4,4′–52						0.01	0.47	0.31	0.07
4–95				0.10	0.08	0.08	15	10	4.1
4′–95				0.07	0.06	0.05	0.16	0.13	0.04
5–95				0.07	0.08	0.08	0.01	0.18	0.17
4,5–95	2.4	0.19	0.03	0.02	0.01	0.03	0.27	0.15	0.12
3–103				0.02	0.01	0.01	0.11	0.07	0.01
4′–101			0.44	0.14	0.16	0.19	1.6	1.8	1.1
3–118	34	30	43	1.3	0.95	1.7	1.5	1.3	3.8
3′–138		1.3	3.8	0.09	0.08	0.22	0.20	0.16	0.54
5–138				0.02	0.01	0.01	0.22	0.07	0.02
3–153		1.8	9.1	0.23	0.15	0.62	3.6	2.2	5.9
4–146					0.01	0.01	0.88	0.29	0.25
3′–180				0.05	0.03	0.09	0.33	0.18	0.32

**panel B: similarity coefficient (cos θ)**									
	brain			liver			serum		
dose group	L	M	H	L	M	H	L	M	H
brain (L)	1	0.88	0.74	0.02	0.02	0.02	0.05	0.05	0.07
brain (M)		1	0.91	0.25	0.25	0.26	0.44	0.45	0.48
brain (H)			1	0.22	0.22	0.23	0.32	0.32	0.39
liver (L)				1	>0.99	>0.99	0.34	0.44	0.68
liver (M)					1	>0.99	0.35	0.44	0.69
liver (H)						1	0.35	0.44	0.69
serum (L)							1	0.99	0.90
serum (M)								1	0.94
serum (H)									1

a Empty values in (A) indicate the nondetectable (below LOD).

The OH-PCB mass profiles reveal interesting differences between the three compartments investigated (Table 1). According to the similarity coefficient cos θ, OH-PCB mass profiles were similar in the liver and serum across various dose groups. In the brain, OH-PCB mass profiles showed some similarities between the 1 mg/kg/d and 6 mg/kg/d exposure groups, while the profiles at 0.1 mg/kg/d differed from those observed at both higher doses. The OH-PCB profiles in the brain differed from those observed in the liver and serum, with cos θ values less than 0.48. These differences are due to the high percentage of 3–28 and 3–118 in the brain and levels of several OH-PCB congeners that are below the detection limit in the brain but not in the liver or serum (Table 1A).

In contrast, 3–28 is only a minor component of the OH-PCB profile in the liver and serum, whereas 3′–28 and 5–28 are major OH-PCB metabolites in the liver and serum based on the average OH-PCB levels. It is possible that 3–28 and 3–118 can more readily penetrate the brain. Although we have shown that PCBs are not oxidized to OH-PCBs in rat hippocampal tissue slice cultures,^38^ local metabolism of PCBs by cytochrome P450 enzymes in the brain^39^ may also explain the higher mass percentage of both metabolites in the brain. In addition to 3–28, several other OH-PCB28 metabolites were detected in the brain at higher PCB doses, including 3–28, 3′–28, 5–28, 2′–28, and 4′–25 (NIH shift product), suggesting that PCB 28 metabolites may play an important but understudied role in the effects of the MARBLES mix on the mouse brain.

### Gene Expression in Different Brain Regions

Changes in the brain transcriptome, particularly in various brain regions, have received less attention following exposure to a PCB mixture. An early study reported brain region-dependent growth-related gene expression changes in the cerebellum and hippocampus of neonatal and juvenile rats exposed developmentally to Aroclor 1254.^40^ Ingenuity Pathway analysis revealed that pathways related to calcium homeostasis, intracellular signaling, axonal guidance, aryl hydrocarbon receptor signaling, and transcripts involved in cell proliferation and differentiation were significantly altered in the hippocampus of these rats.^41^ To expand the available information about the effects of PCBs on the brain transcriptome, RNaseq analyses were used to assess if exposure to the MARBLES mixture altered the transcriptome in the striatum and prefrontal cortex, two brain regions implicated in PCB neurotoxicity.^8,16^

Two bioinformatics tools, iPathwayGuide and gene set analyses (GSA), were used to analyze the RNaseq data based on the number of DEGs for each comparison. The iPathwayGuide bioinformatics tool, which identifies potential pathways contributing to the phenotypes based on the exposure group,^42,43^ was used to compare the high-exposure groups to the controls because of the relatively high number of DEGs. Furthermore, GSA was performed to compare all three exposure groups to the controls. GSA, in which a collection of genes, and not just DEGs associated with specific biological processes, are included in a univariate functional class score, is a popular approach to analyzing RNaseq data. GSA was performed for all comparisons with *clusterProfiler*.^42,43^ GSA has limitations, such as reproducibility and minimal information regarding the biological context of the gene set.^43^

### Single-Cell Deconvolution in the Brain

We assessed if PCB exposure alters cell-type proportions using MuSiC2 for single-cell deconvolution of bulk RNA-seq data.^44^ Briefly, gene counts data from both brain regions were deconvoluted based on the reference data from adult mouse cortical tissue, where bulk results were sorted into seven different cell types found in the brain (astrocytes, endothelial cells, GABAergic neurons, glutamatergic neurons, microglia, oligodendrocytes, and oligodendrocyte precursor cells).^45^ For both brain regions investigated, no significant differences between exposure groups were found in the cell type proportions for any cell type (Figures S1 and S2).

### iPathwayGuide Analysis in the Prefrontal Cortex of PCB-Exposed Mice

**Figure 2:**
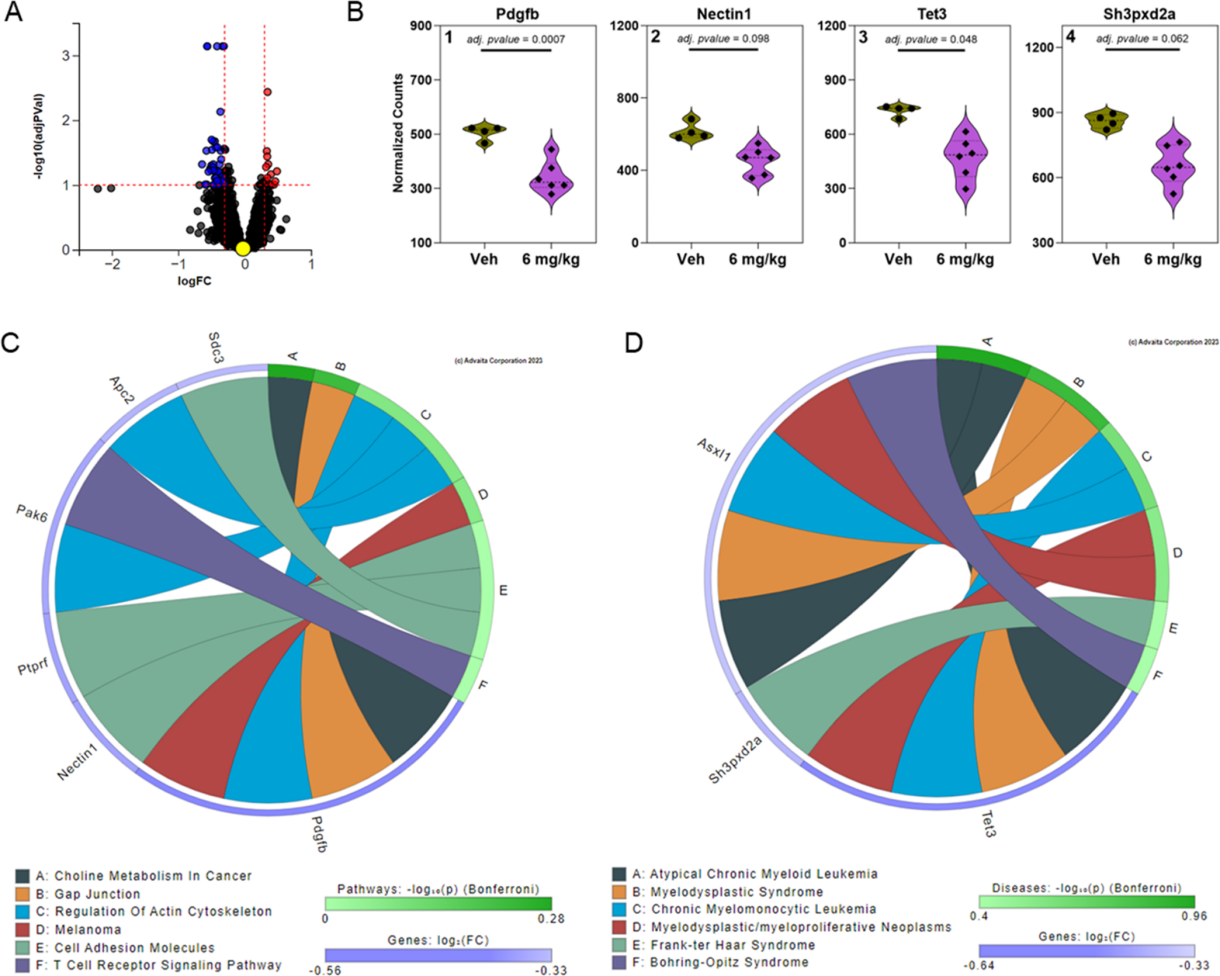
iPathwayGuide analysis from the prefrontal cortex of female mice exposed orally to 6 mg/kg bw/d of the MARBLES mix reveals genes significantly altered and involved in disrupted pathways (i.e., Choline metabolism in cancer) and diseases (i.e., Atypical chronic myeloid leukemia). (A) Volcano plot indicating 66 DEGs based on thresholds of *p*-value <0.1 and log fold change >0.3. The significance is represented in terms of the negative log (base 10) of the *p*-value so that more significant genes are plotted higher on the *y*-axis. The dotted lines represent the thresholds used to select the DE genes: 0.3 for expression change and 0.1 for significance. (B) Significantly altered genes associated with pathways (B1 & B2) and disease (B3 & B4) are shown as violin plots representing the frequency distribution with median and quartiles indicated by dotted lines (bold and fine lines, respectively). (C) KEGG pathway association network based on relationships with significantly altered genes with the top 6 KEGG pathways plotted. (D) The top 6 affected diseases plotted with associations to significantly altered genes. Figures were generated from Advaita Corporation iPathwayGuide.

iPathwayGuide analysis of the gene expression was performed in the prefrontal cortex of mice exposed to 6 mg/kg bw/d MARBLES mix. This analysis revealed 66 DEGs (Figure 2A). Top DEGs included, for example, platelet-derived growth factor subunit B (*Pdgfb*), *Nectin1*, tet methylcytosine dioxygenase 3 (*Tet3*), and SH3 and PX domain-containing protein 2A (*Sh3pxd2a*) (Figure 2B). These DEGs were associated with pathways and diseases altered by PCB exposure. Interestingly, oral exposure to the MARBLES mix affected *Mapt* expression in the prefrontal cortex in a dose-dependent manner. (Figure S3). *Mapt* is implicated in the manifestation of frontotemporal dementias such as Alzheimer’s Disease within humans,^46^ and *Mapt* expression is impacted by endocrine disrupting chemicals such as PCBs.^47^

The iPathwayGuide analysis identified pathways, for example, “choline metabolism in cancer,” “gap junction,″ and “cell adhesion molecules” that were affected by PCB exposure in the prefrontal cortex (Figure 2C). Several KEGG pathways, including “choline metabolism in cancer”, “gap junction”, and “melanoma”, were associated with *Pdgfb* expression, with a decreased expression following PCB exposure. Changes in the “gap junction” pathway are consistent with findings that gap junctions and the permeability of the blood-brain barrier are altered by PCB exposure *in vitro*(48) and *in vivo*.^49,50^ According to the disease ontology analysis, several genes, including *Tet3*, were associated with inflammatory diseases broadly categorized as “myelodysplastic/myeloproliferative neoplasms” in the prefrontal cortex (Figure 2D). *Tet3* is a regulator of mitochondrial respiration in a Neuro2A mouse neuroblastoma cell line^51^ and an epigenetic regulator of cell fate in neuronal precursors.^52^ Because PCB exposure alters DNA methylation in the brain of mice developmentally exposed to the MARBLES mix,^53^ further studies of the role of *Tet3* in PCB neurotoxicity are warranted.

Mitochondrial dysfunction plays an important role in neurodevelopmental and neurodegenerative disorders; however, there is limited evidence that mitochondrial dysfunction plays a role in PCB neurotoxicity.^54^ A recent study on astrocytes in culture reported a loss of mitochondrial membrane potential, changes in mitochondrial structure, and impaired mitochondrial function after exposure to PCB52 and its human-relevant metabolites.^55^ In the brain of PND14 rat offspring developmentally exposed to Aroclor 1254 via the maternal diet, differential protein expression related to energy metabolism in mitochondria, such as ATP synthase, subunit β (ATP5B), creatine kinase, and malate dehydrogenase was induced.^56^*In vitro* results also demonstrate that PCBs adversely impact mitochondria function in neuroblastoma cells.^57^ Finally, a study in zebrafish brains showed disruption of energy homeostasis that could be explained by impaired transcriptional pathways of mitochondrial function and lipid metabolism regulation following exposure to an environmentally relevant mixture of PCBs and polybrominated diphenylethers.^58^

### Gene Set Analysis in the Prefrontal Cortex of PCB-Exposed Mice

**Figure 3:**
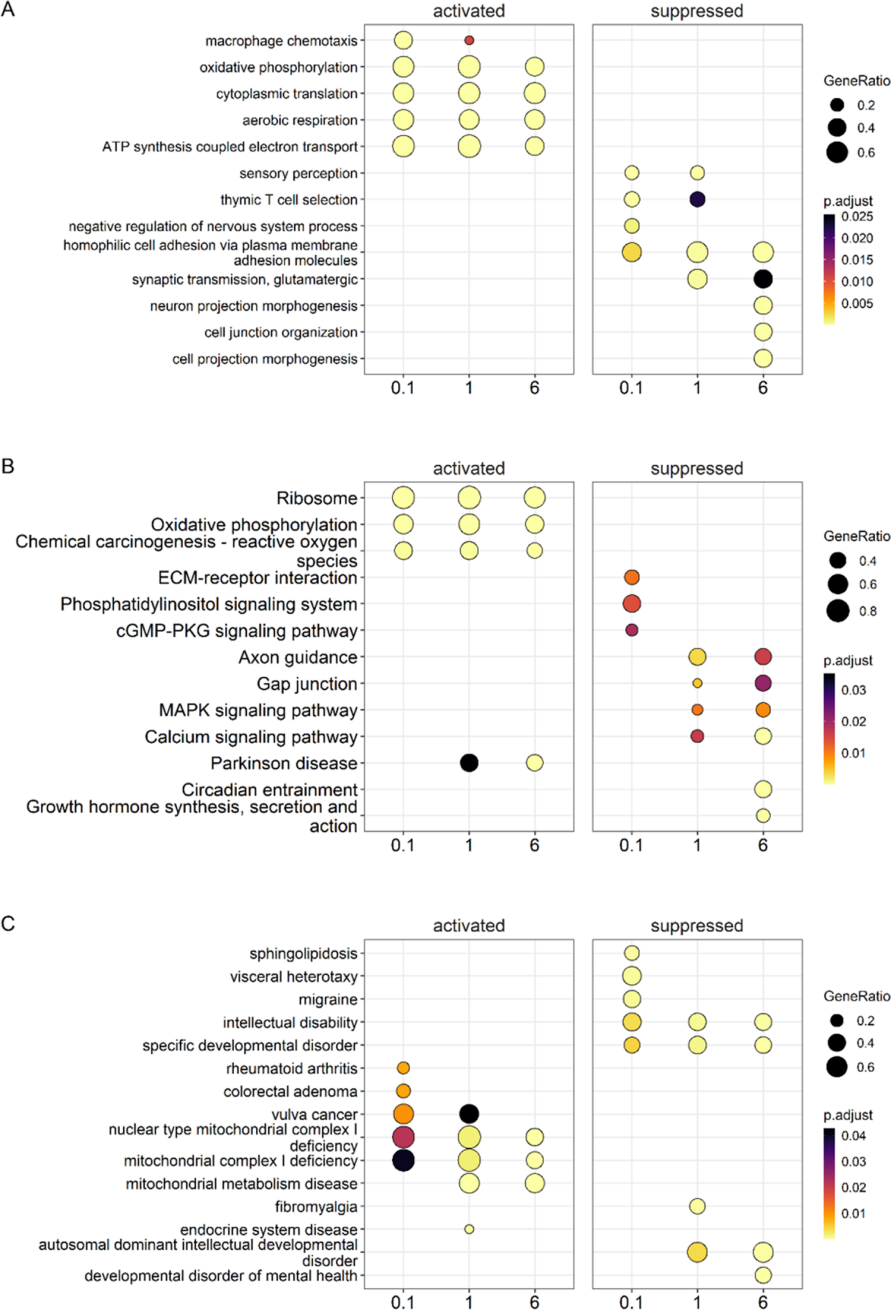
Gene set analysis comparison conducted for prefrontal cortex samples of females orally exposed to the MARBLES mix. (A) Gene enrichment dot plot of top 3 activated and suppressed genes from each exposure group to identify overlapping results. For example, several processes such as “oxidative phosphorylation”, “cytoplasmic translation”, and “aerobic respiration” were significantly activated while one processes, “homophilic cell adhesion”, was suppressed. (B) KEGG pathway enrichment dot plot of top 3 activated and suppressed pathways across exposure groups reveals “oxidative phosphorylation” is significantly activated and pathways such as “axon guidance”, “gap junction”, and “calcium signaling” were significantly suppressed in the two highest exposure groups. (C) A disease enrichment analysis identifying the top 3 activated and suppressed diseases. A shared activated disease across all exposure groups is “mitochondria complex 1 deficiency” which echos the oxidative phosphorylation KEGG pathway results. The suppressed diseases for all exposures included both “intellectual disability” and “specific developmental disorder”. The color indicates the adjusted *p*-values of the estimated significance of the corresponding enrichment analysis. The dot size indicates GeneRatio or the number of genes in a particular gene set enriched over the total number of genes in the gene set, KEGG pathway, or disease ontology based on the KEGG database. The *x*-axis indicates dosing groups. Figures were generated using R packages *fgsea* and *clusterProfiler.*.

Gene enrichment analysis identified processes activated in the prefrontal cortex by PCB exposure, including “oxidative phosphorylation,” “cytoplasmic translation,” and “aerobic respiration” (Figure 3A). Processes suppressed in mice dosed with the MARBLES mix included, for example, “homophilic cell adhesion”. Like the gene enrichment analysis, KEGG pathway analysis showed that the “oxidative phosphorylation” pathway was activated across all three exposure groups. Pathways suppressed by PCB exposure included, for example, the “axon guidance” and “gap junction” pathways, two pathways critical for the proper development and functioning of the brain (Figure 3B).^59,60^ In the disease enrichment analysis, exposure to PCBs was found to suppress diseases, such as “intellectual disability” and “specific developmental disorder,″ in the prefrontal cortex (Figure 3C). Moreover, all exposure groups showed significant activation of “mitochondria complex 1 deficiency”. Thus, consistent with the iPathwayGuide results discussed above, the gene set analysis results suggest that PCB exposure affects the mitochondria and gap junction function in the prefrontal cortex. The suppression of “intellectual disability” and “specific developmental disorder” in the disease enrichment analysis contrasts with the established developmental neurotoxicity of PCBs.^7,8,61^ One possible explanation for our finding is that PCBs may disrupt specific biochemical pathways differently across various life stages.

### iPathwayGuide Analysis in the Striatum of PCB-Exposed Mice

**Figure 4:**
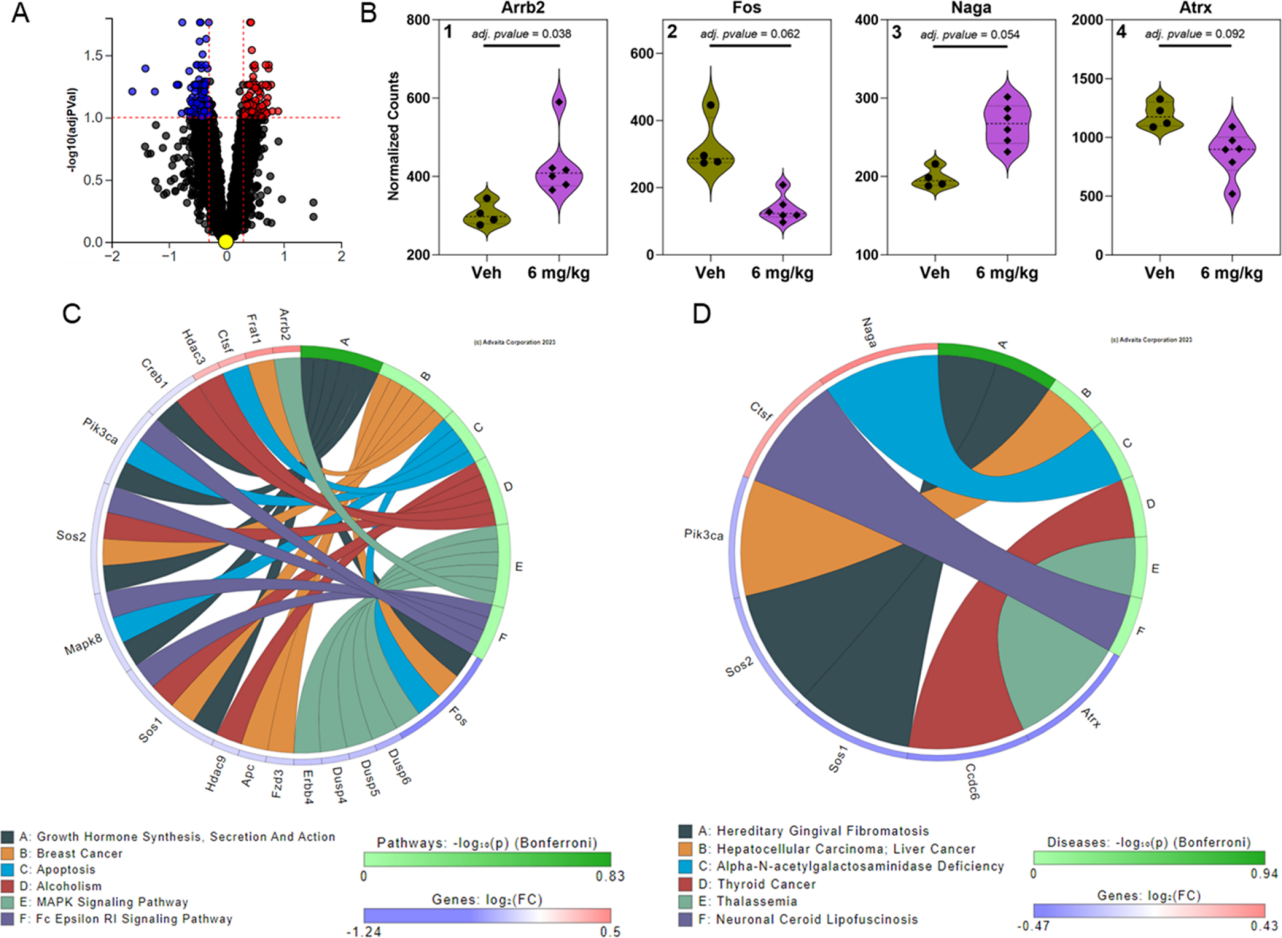
iPathwayGuide analysis from the striatum of female mice exposed orally to 6 mg/kg bw/d of the MARBLES mix reveals genes significantly altered and involved in disrupted pathways (i.e., Hereditary gingival fibromatosis) and diseases (i.e., Growth hormone synthesis). (A) Volcano plot indicating 302 DEGs based on thresholds of *p*-value <0.1 and log fold change >0.3. The significance is represented in terms of the negative log (base 10) of the *p*-value so that more significant genes are plotted higher on the *y*-axis. The dotted lines represent the thresholds used to select the DE genes: 0.3 for expression change and 0.1 for significance. (B) Significantly altered genes associated with pathways (B1 & B2) and disease (B3 & B4) are shown as violin plots representing the frequency distribution with median and quartiles indicated by dotted lines (bold and fine lines, respectively). (C) KEGG pathway association network based on relationships with significantly altered genes with the top 6 KEGG pathways plotted. (D) The top 6 affected diseases plotted with associations to significantly altered genes. Figures were generated from Advaita Corporation iPathwayGuide.

iPathwayGuide pathway analysis in the striatum of mice exposed to 6 mg/kg bw/d of the MARBLES mix revealed 302 genes significantly altered by PCB exposure, indicating that the striatum may be more susceptible than the prefrontal cortex to gene expression changes following PCB exposure (Figure 4A). Examples of DEGs in the striatum include arrestin β 2 (*Arrb2*), Fos proto-oncogene, AP-1 transcription factor subunit (*Fos*), α-*N*-acetylgalactosaminidase (*Naga*), and the transcriptional regulator ATRX (*Atrx*) (Figure 4B). A link to PCB neurotoxicity has not been established for these DEGs; however, these genes are implicated in neurodevelopmental or neurodegenerative diseases. For example, *Arrb2* has been implicated in Alzheimer’s disease.^62^ Overexpression of *Atrx*, an epigenetic regulator, results in neurodevelopmental defects in mice,^63^ and ATRX mutations cause a human neurodevelopmental syndrome.^64^ The genes in Figure 4B were associated with several pathways and diseases (Figure 4C,D). For example, a decrease in FOS expression due to PCB exposure was associated with the “growth hormone synthesis, secretion and action,” “apoptosis,” and “MAPK signaling” pathways. These results are consistent with earlier studies demonstrating that PCBs trigger apoptosis and activate MAPK signaling pathways to stimulate dendritic arborization.^65−67^ Several diseases identified in the disease ontology analysis are associated with *Naga* (Figure 4D). Deficiencies in this gene are implicated in neurological manifestations that resemble autism spectrum disorders.^68^ Therefore, the increased expression of *Naga* (Figure 4B) may compensate for PCB-induced damage in the striatum.

### Gene Set Analysis in the Striatum of PCB-Exposed Mice

**Figure 5:**
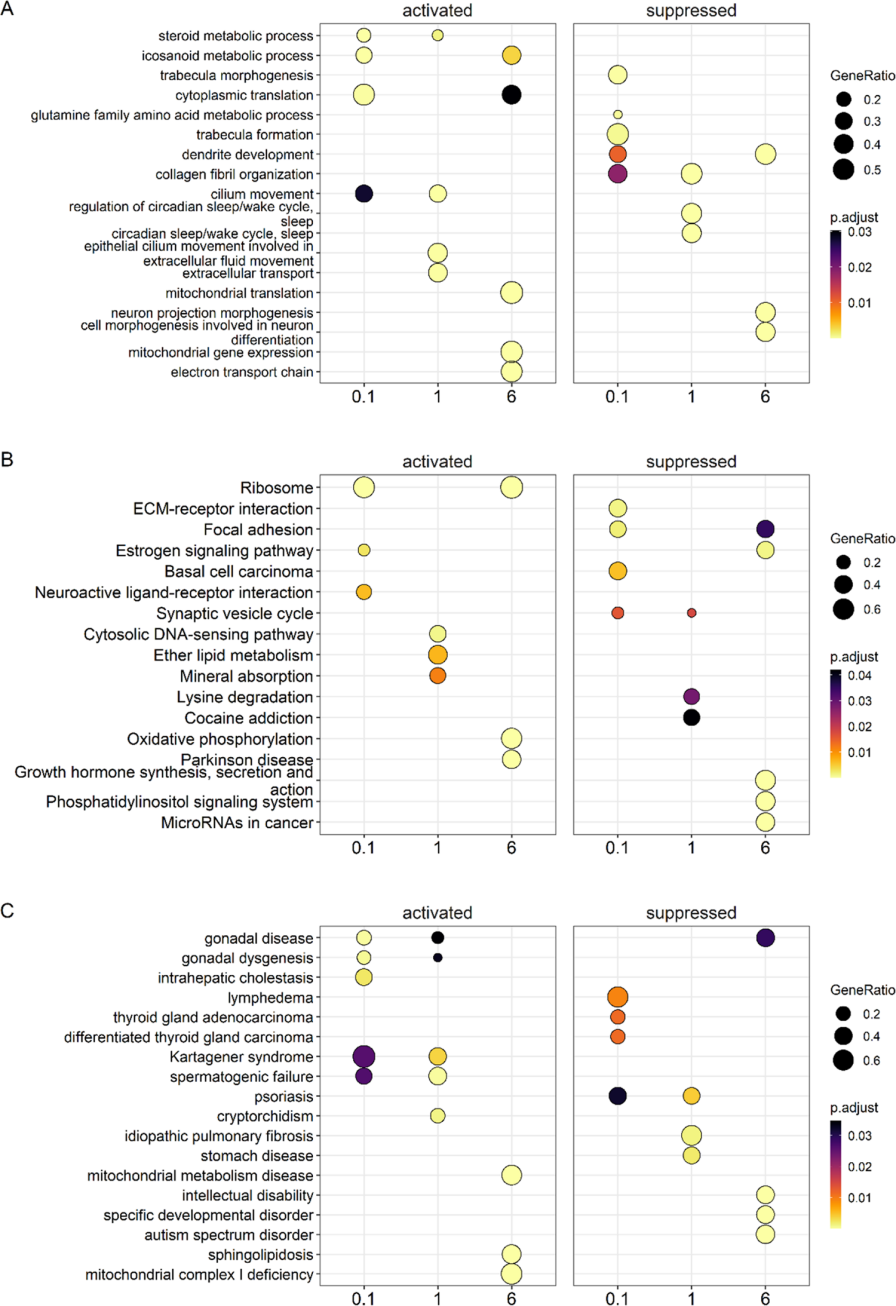
Gene set analysis comparison conducted for striatum samples of females orally exposed to the MARBLES mix. (A) Gene enrichment dot plot of top 3 activated and suppressed genes from each exposure group to identify overlapping results. For example, “cytoplasmic translation” was significantly activated while “dendrite development” was significantly suppressed in two out of the three exposure groups. (B) KEGG pathway enrichment dot plot of top 3 activated and suppressed pathways across exposure groups reveals that only one pathway result was significantly activated, overlapping in two groups, the “ribosome” KEGG pathway. For suppressed pathways, “focal adhesion”, and “synaptic vesicle cycle”, were the only two pathways overlapping in two of the exposure groups. (C) A disease enrichment analysis identifying the top 3 activated and suppressed diseases. A shared activated disease across two exposure groups is “gonadal disease” while the only suppressed disease shared by two groups was “psoriasis”. The color indicates the adjusted *p*-values of the estimated significance of the corresponding enrichment analysis. The dot size indicates GeneRatio or the number of genes in a particular gene set enriched over the total number of genes in the gene set, KEGG pathway, or disease ontology based on the KEGG database. The *x*-axis indicates dosing groups. Figures were generated using R packages *fgsea* and *clusterProfiler.*.

In the gene enrichment analysis, genes related to “cytoplasmic translation” were significantly activated. Genes involved in “dendrite development” were significantly suppressed in two of the three exposure groups, suggesting that PCB exposure decreases dendritic arborization (Figure 5A). However, PCBs have consistently been shown to increase dendritic arborization in rodents exposed to PCBs throughout gestation and lactation, as reviewed previously.^8,61^ Differences in the age of the mice and PCB exposure paradigm may explain these conflicting results.^69^ Moreover, changes in gene expression pathways do not necessarily translate into morphological differences, such as dendritic arborization. Only one activated pathway, the “ribosome” KEGG pathway, was observed in more than one exposure group. This pathway is involved in the production and assembly of ribosomes,^70^ suggesting that PCB exposure affects protein homeostasis in the striatum. The “focal adhesion” pathway was suppressed in the low and high PCB exposure group (Figure 5B). While these pathways are not associated with adverse outcomes following PCB exposure, they are more broadly linked to adverse neurological outcomes.^71−73^

### Cytochrome P450 (CYP) Gene Expression in the Striatum and Prefrontal Cortex

Because CYPs expressed in the mouse brain may result in the local formation of neurotoxic metabolites,^39,74^ thus contributing to neurotoxic outcomes, we investigated the effect of oral exposure to the MARBLES mix on the expression of CYPs in the mouse brain compared to hepatic CYP expression (Figure S4). Based on the RNaseq data, *Cyp2a5, Cyp2s1*, and *Cyp4x1* were expressed at low levels in the two brain regions investigated. PCB exposure did not affect the expression of these three CYPs. Moreover, CYPs implicated in the hepatic metabolism of PCBs, including *Cyp1a2, Cyp2b10, Cyp2c50*, and *Cyp3a41a* enzymes,^75^ had low (<10 counts) or no expression in the two brain regions investigated. This observation is consistent with a study of adult male mice that did not observe *Cyp2b10* or *Cyb1a2* expression in the cerebellum.^76^ In contrast to the brain, *Cyp2b10* and *Cyp2c50* expression increased with the dose in the livers of PCB-exposed mice. Similar trends in the hepatic expression of both CYPs have been observed in mice exposed to the Fox River PCB Mixture, consistent with the well-established, constitutive androstane receptor (CAR)-mediated induction of these CYPs by PCBs.^77^ These findings do not support the hypothesis that the OH-PCBs detected in the brain are formed by localized oxidation of PCB in the brain.

### Metabolomics in the Striatum

The effects of PCB exposure on the metabolome are typically studied in serum. For example, endogenous metabolites in serum or their corresponding pathways affected by PCB exposure include linoleic acid metabolism, glycerophospholipids, and sphingolipids.^78−80^ In contrast, the brain metabolome in mice exposed to PCBs is relatively understudied.^81^ Therefore, we investigated dose-dependent changes in the metabolome of female mice exposed to the MARBLES mix using targeted analyses of water-soluble metabolites. Hierarchical cluster analysis of water-soluble metabolites showed no robust clustering of samples (Figure S5), and no significantly altered metabolites were observed in this brain region in PCB-exposed mice, irrespective of the dose. Likely, the metabolomic analysis did not capture localized, cell-type-specific effects of PCB exposure on the metabolome, a possibility that requires further attention.

### Gene Expression in the Liver

**Figure 6:**
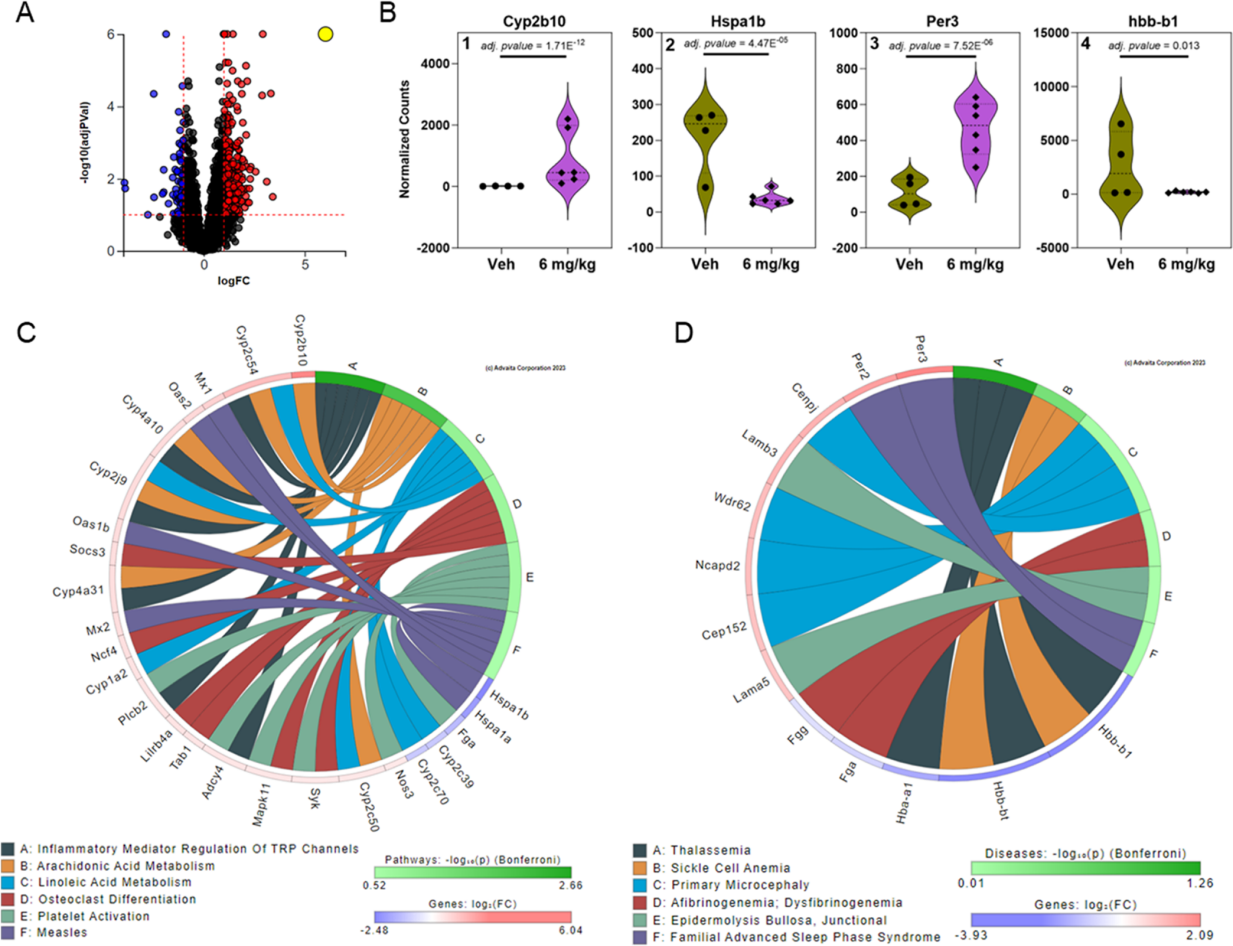
iPathwayGuide analysis from the liver of female mice exposed orally to 6 mg/kg bw/d of the MARBLES mix reveals genes significantly altered and involved in disrupted pathways (i.e., inflammatory mediator regulation of TRP channels) and diseases (i.e., Thalassemia). (A) Volcano plot indicating 491 DEGs based on thresholds of *p*-value <0.1 and log fold change >1. The significance is represented in terms of the negative log (base 10) of the *p*-value so that more significant genes are plotted higher on the *y*-axis. The dotted lines represent the thresholds used to select the DE genes: 1 for expression change and 0.1 for significance. (B) Significantly altered genes associated with pathways (B1 & B2) and disease (B3 & B4) are shown as violin plots representing the frequency distribution with median and quartiles indicated by dotted lines (bold and fine lines, respectively). (C) KEGG pathway association network based on relationships with significantly altered genes with the top 6 KEGG pathways plotted. (D) The top 6 affected diseases plotted with associations to significantly altered genes. Figures were generated from Advaita Corporation iPathwayGuide.

Because PCB-mediated effects on the liver may indirectly affect brain health via the liver-brain axis,^82^ we also explored how the PCBs present in the liver affect the liver transcriptome. Overall, the liver transcriptome appeared to be more impacted by PCB exposure than the brain (Figure S6). For example, the iPathwayGuide analysis identified 491 genes significantly altered in the liver of the 6 mg/kg bw/d PCB exposure group compared to vehicle controls (Figure 6A). No overlap in the DEGs was observed between the prefrontal cortex, striatum, and liver at the high PCB dose (Figure S7), consistent with tissue and brain region-specific differences in the regulation of gene expression, as described above for CYPs. Seven hepatic DEGs were affected by PCB exposure across all three dose groups (Figures S8, S9).

**Figure 7:**
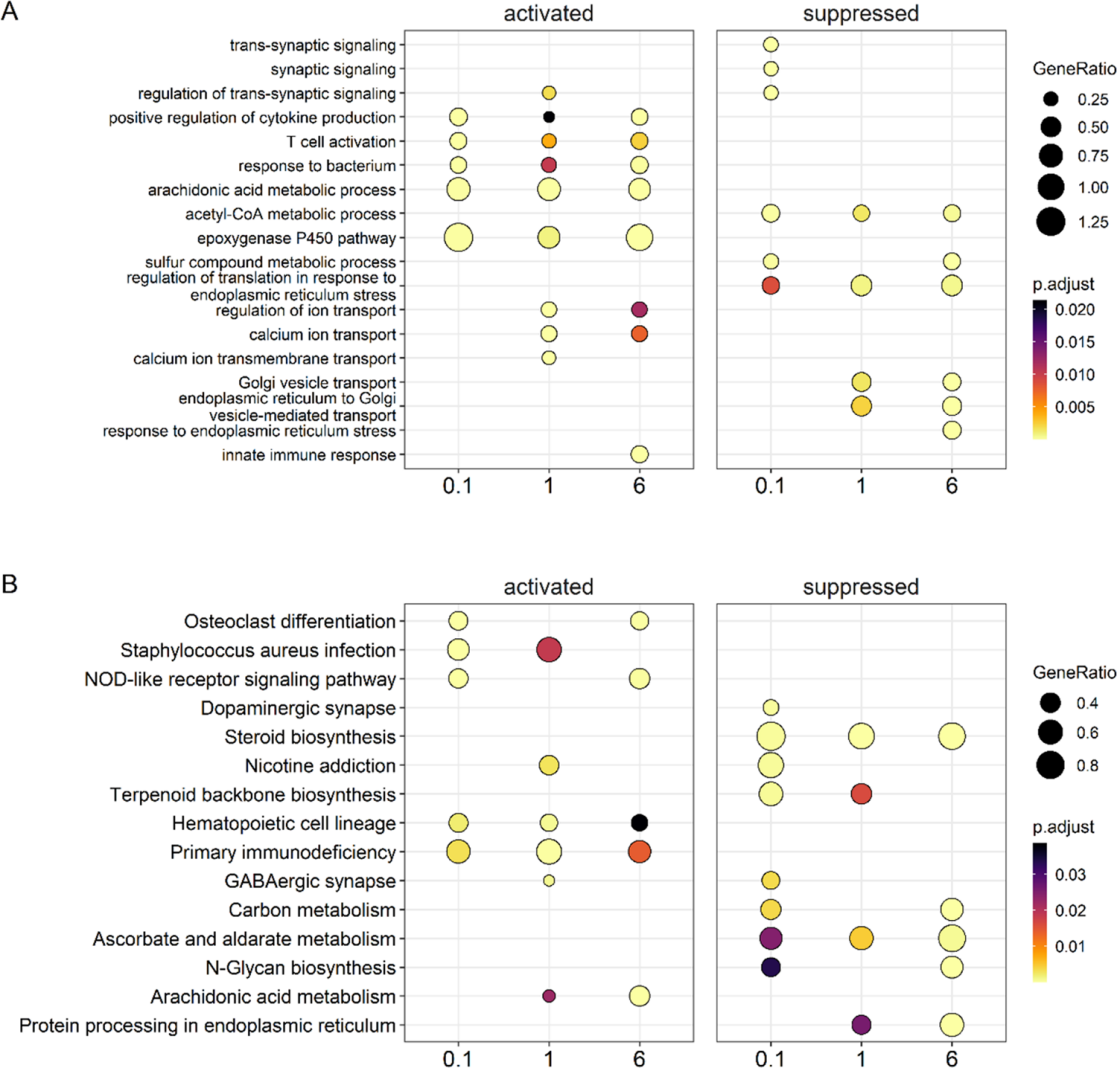
Gene set analysis comparison was conducted for liver samples of females orally exposed to the MARBLES mix. (A) Gene enrichment dot plot of top 3 activated and suppressed genes from each exposure group to identify overlapping results. For example, “T cell activation” is significantly activated while “regulation of translation in response to endoplasmic reticulum stress” is suppressed. (B) KEGG pathway enrichment dot plot of top 3 activated and suppressed pathways across exposure groups reveals “primary immunodeficiency” is significantly activated and “steroid biosynthesis” is suppressed. The color indicates the adjusted *p*-values of the estimated significance of the corresponding enrichment analysis. The dot size indicates GeneRatio or the number of genes in a particular gene set enriched over the total number of genes in the gene set, KEGG pathway, or disease ontology based on the KEGG database. The *x*-axis indicates dosing groups. Figures were generated using R packages *fgsea* and *clusterProfiler.*.

In the iPathwayGuide analysis, the top DEGs associated with the pathways and diseases altered by PCB exposure in the liver are shown in Figure 6B. Expression of *Cyp2b10*, a cytochrome P450 enzyme likely involved in the metabolism of PCBs, increased in a dose-dependent manner. *Cyp2b10* was associated with KEGG pathways related to metabolism, such as “arachidonic acid metabolism” (Figure 6C). The disease ontology analysis, period circadian regulator 3 (*Per3*), a gene involved in period circadian proteins, was associated with “familial advanced sleep phase syndrome” (Figure 6D). Results from gene enrichment analysis across all exposure groups also revealed activation of several metabolic processes, such as the “arachidonic acid metabolic process” following PCB exposure (Figure 7A). The “primary immunodeficiency” pathway was significantly activated for all exposure groups in the pathway enrichment analysis (Figure 7B). Possible implications of these changes in the liver transcriptome include systemic effects on the polyunsaturated fatty acids homeostasis, circadian rhythm, and immune system that may contribute to neurotoxic outcomes.

### Network Analysis

Network analysis using xMWAS^83^ was performed to integrate the PCB tissue levels and transcriptomic data from the prefrontal cortex and the striatum. These network analyses provide a systems-level understanding of the interactions of specific PCBs or OH-PCBs and the dysregulation of specific DEGs. This analysis unveiled novel relationships between individual PCB and OH-PCB congeners and particular genes altered by PCB exposure in the brain that were not apparent in the conventional gene set and pathway analysis approaches and require further attention to fully characterize the effects of PCB exposure on the brain transcriptome. Results from an analogous analysis in the liver are presented in Figure S10.

### Network Analysis in the Prefrontal Cortex

**Figure 8:**
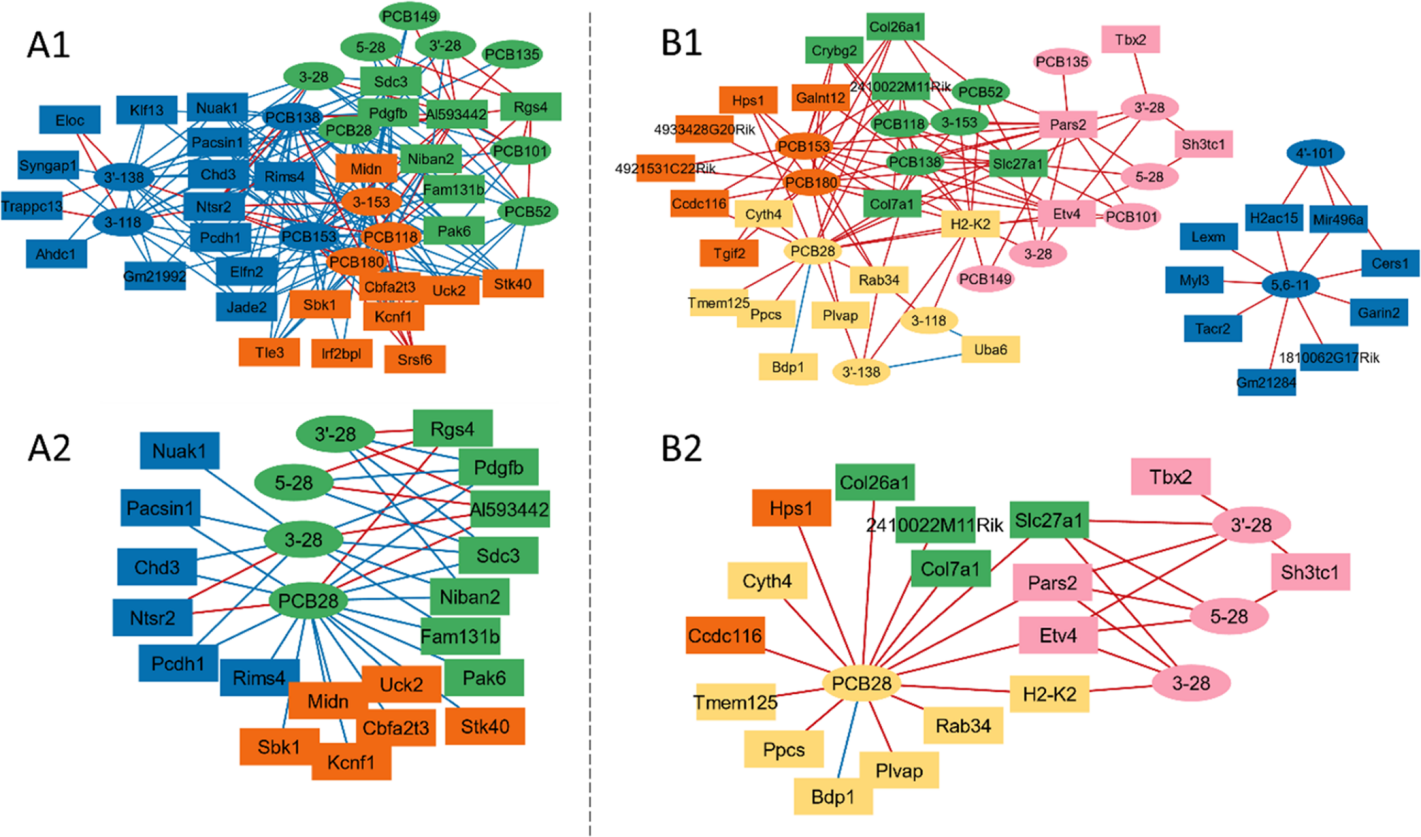
Interaction network analyses of brain PCB and OH-PCB levels and the transcriptome in (A) the prefrontal cortex and (B) the striatum identify three and five clusters, respectively. Panels show (A1) the full network in the prefrontal cortex, (A2) the subnetwork of PCB28 and its OH-metabolites in the prefrontal cortex, (B1) the full network in the striatum, and (B2) a subnetwork of PCB28 and its OH-metabolites in the striatum. Network analyses were performed with xMWAS (version 0.552)^83^ using a threshold of absolute correlation coefficients >0.7 for the prefrontal cortex, > 0.75 for the striatum, and *p* < 0.05. Nodes in the same cluster share the same color. The node shape represents PCBs (ovals) and genes (rectangles). The edge color indicates positive (red) and negative (blue) correlations.

Network analysis of brain PCB levels and DEGs across all exposure groups identified three clusters in the prefrontal cortex (Figure 8A1). Thirty transcripts and 15 PCB and OH-PCB congeners had significant correlations in the network (**|***r*|> 0.7, *p* < 0.05) (Figure 8A1). *Pdgfb*, which is associated with “choline metabolism in cancer”, “gap junction”, “regulation of actin cytoskeleton”, and “melanoma” in the iPathwayGuide analyses (Figure 2C), and syndecan 3 (*Sdc3*) were negatively correlated with most PCBs (14 out of 15 PCB congeners in the network). Conversely, *Al593442* showed a positive correlation with 13 PCBs and OH-PCBs. A subnetwork focusing on PCB28 is shown in Figure 8A2. *Pdgfb* and *Sdc3* negatively and *Al593442* positively interacted with PCB28 and its metabolites. Interestingly, *Sdc3*, a pleiotrophin receptor highly expressed by nigral dopaminergic neurons, regulates dopaminergic neurons and may be involved in Parkinson’s disease.^84^ Additionally, *Pdgf* signaling plays a role in brain function, regulating synaptic plasticity and function.^85^ These results indicate that *Pdgfb* and *Sdc3* are novel targets of PCB28 in the prefrontal cortex.

### Network Analysis in the Striatum

Network analysis of brain PCB levels and DEGs across all exposure groups identified five clusters in the striatum (Figure 8B1). Thirty-two genetic transcripts and 17 PCB and OH-PCB congeners had significant correlations in the network (**|***r*|> 0.75, *p* < 0.05). All identified genes were positively correlated to PCBs, except B double prime 1 (*Bdp1*) and ubiquitin like modifier activating enzyme 6 (*Uba6*). Histocompatibility 2, K region locus 2 (H2-K2), which is part of the major histocompatibility complex (MHC) class I gene, is implicated in the functioning of the immune system, particularly in antigen presentation and immune surveillance,^86^ was positively interconnected with most PCB and OH-PCB congeners. In addition, H2-K2 and H2-D2 play a role in synaptic plasticity and motor learning.^87^ Although the effects of PCB exposure on H2-K2 and H2-D2 gene expression have not been reported, changes in the immune system gene expression are consistent with the overall pro-inflammatory effects of PCB exposure.^88^

A subnetwork showing the DEGs that correlated with PCB28, the PCB congener with the highest levels in the brain, and its metabolites is depicted in Figure 8B2. Three genes, including solute carrier family 27 member 1 (*Slc27a1*), prolyl-tRNA synthetase 2 (*Pars2*), and ETS variant transcription factor 4 (*Etv4*), were positively correlated with PCB28 and three of its metabolites, including 3–28, 3′–28 and 5–28. *Slc27a1* is a gene that encodes a fatty acid transport protein involved in the transport of long-chain fatty acids.^89^ Long-chain fatty acids are crucial for many cellular processes, including energy metabolism, intracellular signal transduction, and membrane synthesis.^90^ Dysregulation of fatty acid metabolism in the brain has been implicated in neurodegenerative diseases.^91^ The *Pars2* gene encodes mitochondrial methionyl-tRNA synthetase, an enzyme involved in mitochondrial protein synthesis. This enzyme is crucial for the function of mitochondrial respiratory complexes in oxidative phosphorylation.^92^*Etv4* gene encodes a transcription factor protein that plays a key function in the progression of many cancers.^93^ While the direct role of *Etv4* in the striatum is less studied, it regulates the growth and arborization of pyramidal cell dendrites in the development and plasticity of the hippocampus.^94^ Overall, the subnetwork analysis identified *Slc27a1*, *Pars2*, and *Etv4*, genes essential for cellular processes in the striatum, as novel targets for PCB28 and its hydroxylated metabolites that require further investigation.

## Conclusions

The study investigates dose-dependent and brain region-specific transcriptomic effects of polychlorinated biphenyls (PCBs) in female mice exposed to the human-relevant MARBLES mix. All PCB congeners in the MARBLES mix and their hydroxylated metabolites were present in the tissues investigated, with notable differences in the tissue profiles due to the rapid elimination of PCB11 and the accumulation of PCB28. In the prefrontal cortex, PCB exposure activated oxidative phosphorylation pathways while suppressing axon guidance pathways, consistent with recent findings reporting the effects of PCBs and their metabolites on mitochondria in astrocytes in culture.^36^ In the striatum, significant changes in genes associated with neurodevelopmental and neurodegenerative diseases were observed, with pathways related to growth hormone synthesis and dendrite development affected following exposure to the MARBLES mix. The liver showed considerable activation of metabolic processes and, unlike the two different brain regions, induction of drug-metabolizing enzymes (e.g., *Cyp2b10*), highlighting tissue-specific responses to PCB exposure that may indirectly impact brain health. Network analysis revealed complex interactions between individual PCBs and OH-PCBs with DEGs. Subnetwork analyses identified *Pdgfb* and *Sdc3* as novel targets of PCB28 in the prefrontal cortex, and *Slc27a1*, *Pars2*, and *Etv4* as novel targets of PCB28 and its hydroxylated metabolites in the striatum. These findings underscore the significance of employing systems biology approaches to elucidate the relationships between individual PCB congeners, their metabolites, and the corresponding alterations in transcriptome, proteome, metabolome, and epigenome across different brain regions. Moreover, single-cell and spatial transcriptomic studies are needed to pinpoint the effects of individual PCBs or their metabolites on specific cell populations in different brain regions. Identifying these relationships will significantly advance our understanding of PCB-induced neurotoxicity.

## Materials and Methods

### Chemicals

The PCB congeners (PCB11, 28, 52, 84, 95, 101, 118, 135, 138, 149, 153, and 180) used to make the MARBLES mix were synthesized and authenticated as reported earlier (Figure S11).^10,95^ The PCB nomenclature is based on the US EPA Table of PCB congeners.^96^ The nomenclature of OH-PCBs is an abbreviated version of the PCB metabolite nomenclature,^97^ where the first number indicates the position of the OH-group on the biphenyl moiety, and the second number reflects the number of the corresponding PCB congener. The abbreviations and unique identifiers of the analytical OH-PCB standards are summarized in Table S4. For additional details regarding the analytical standards, see the Supporting Information.

### Animal Exposure

This study used tissues from female mice exposed to the MARBLES mix as part of a larger study of the developmental neurotoxicity of the MARBLES mix.^10,11^ The MARBLES mix contains PCB11 (24.3%), PCB28 (48.2%), PCB52 (4.5%), PCB84 (1.5%), PCB95 (1.2%), PCB101 (4.5%), PCB118 (4.9%), PCB135 (1.3%), PCB138 (1.7%), PCB149 (2.1%), PCB153 (3.1%), and PCB180 (2.8%). This PCB mixture approximates the PCB profile identified in serum from pregnant women enrolled in the MARBLES cohort.^10,11^ All experimental procedures involving animals were reviewed and approved by the University of California Davis IACUC (Institutional Animal Care and Use Committee; Protocol #20584 approved August 2018) and conform with the National Research Council’s Guide for the Care and Use of Laboratory Animals. The data from this project are freely available on Iowa Research Online at 10.25820/data.007310.^98^

Female C57Bl/6J mice (>6-week-old) were randomized into exposure groups and orally exposed to 0 (*n* = 4), 0.1 (*n* = 5), 1 (*n* = 6), or 6 mg/kg bw/d (*n* = 6) of the MARBLES mix in organic peanut butter (Trader Joe’s, Monrovia, California)/organic peanut oil (Spectrum Organic Products, Melville, New York) for 7 weeks, as described previously.^9^ Based on an earlier study, exposure to 6 mg/kg/d via the maternal diet results in PCB brain levels in weanling rats that are comparable to human PCB levels in the brain.^15^ Animals were singly housed in clear plastic cages with corncob bedding while maintained on a 12 h light and dark cycle at 22 ± 2 °C and 40–50% humidity. Food (Diet 5058, LabDiet, Saint Louis, Missouri) and water were available *ad libitum*.^9^ Approximately 20 h after the final PCB exposure, mice were euthanized with CO_2_, quickly followed by blood collection via cardiac puncture, and transcardially perfused with a peristaltic pump and cold (4 °C) PBS to remove blood from the brain tissue for analysis, and target tissues were rapidly excised. Samples were stored at −80 °C until further analysis. A summary of the pre- and postexposure bodyweights is shown in Figure S12.

### PCB and OH-PCB Extraction from the Brain, Liver, and Serum

*For safety reasons, proper training and personal protective equipment are required when handling PCBs, group 1 human carcinogens, and diazomethane, a toxic and explosive derivatization reagent*. PCBs and OH-PCBs were extracted using liquid–liquid extraction protocols.^99,100^ Briefly, about 100 mg of brain (102 ± 11 mg, *n* = 25, including samples for the extraction of the ongoing recovery and precision [OPR] standard) and 200 mg of liver (207 ± 48 mg, *n* = 23, including samples for the extraction of the ORP standard) were homogenized with 3 mL of 2-propanol using a TissueRuptor (QIAGEN, Hilden, Germany) followed by adding 10 ng PCB (PCB15 and PCB117 in isooctane) and 10 ng OH-PCB (4′–9, 4–91, and 4′–159 in methanol) as surrogate standards to all samples. Samples were then extracted with diethyl ether and hexane (1:9, v/v), followed by 5 mL of 0.1 M phosphoric acid in 0.9% sodium chloride solution. The organic extracts were concentrated under a gentle nitrogen stream and derivatized with diazomethane in diethyl ether at 4 °C overnight.^99^ Next, extracts were cleaned up by base solution (1 M KOH in 95% ethanol) at elevated temperature (50 °C) for 1 h, and then passed through a sulfuric acid and silica gel (1:5, w/w) cartridge for lipid removal. Finally, the extracts were concentrated under a gentle nitrogen stream, and the internal standards (d-PCB30 and PCB204) were added to each sample before GC-MS/MS analysis.

Serum (92 ± 24 mg, *n* = 24) samples were extracted similarly to the tissues, with modifications. Briefly, 1 mL of 6 M HCl was added to the serum after homogenization. After adding the surrogate standards, PCB and OH-PCB were extracted in 2-propanol and hexane: methyl *tert*-butyl ether (1:1, v/v), followed by washing with 3 mL of 1% KCl. The extracts were then concentrated and derivatized as described for tissue samples. After further cleanup using 2-propanol and tetrabutylammonium hydrogen sulfate, the extracts were subjected to the same cleanup steps described above for tissue samples.

### GC-MS/MS Analysis

A GC-MS/MS system (Agilent 7890B GC system, Agilent 7000D Triple Quad, Agilent 7693 autosampler; Agilent, Santa Clara, California, United States) equipped with an SPB-Octyl capillary column (50% n-octyl/50% methyl siloxane, 30 m × 0.25 mm ID, 0.25 μm film thickness; Supelco, Bellefonte, Pennsylvania) was used for PCB and OH-PCB metabolite quantification. The system used helium as the carrier gas (flow rate of 0.8 mL/min) and nitrogen as the collision gas. The solvent vent injection mode was used for the sample injections, with an initial temperature of 45 °C, an initial time of 0.06 min, a ramp of 600 °C/min to an inlet temperature of 325 °C at 5 psi. The GC oven temperature program was 45 °C for 2 min, 45 to 75 °C at 100 °C/min, hold for 5 min, 75 to 150 °C at 15 °C/min, hold for 1 min, 150 to 280 at 2.5 °C/min, and hold 5 min. The triple quadrupole electron ionization source was set to 280 °C. A list of precursor ions, product ions, and collision energies for each analyte is provided in Table S5. Details regarding the quality assurance and quality control are described in the Supporting Information (Tables S6–S8). PCB and OH-PCB levels were adjusted for the recoveries of the appropriate recovery standard and are reported relative to the tissue wet weight (Table S1). Congener profiles were compared using the pairwise similarity coefficient cos θ, with cos θ = 1 indicating identical and cos θ = 0 indicating different congener profiles.^101^

### RNA Sequencing and Analysis

Total RNA was isolated from the striatum, prefrontal cortex, and liver following manufacturer’s instructions and checked for purity with a nanodrop (Thermo Fisher Scientific, Fair Lawn, New Jersey). Samples with RNA integrity number (RIN) ≥ 8.0 were submitted to Novogene (Davis, California) for Illumina RNA sequencing. For additional details regarding the RNA extraction and RNA sequencing, see the Supporting Information. Raw fastq files are deposited in the Gene Expression Omnibus database at GSE252621 (access token: mxwluquazrippct).

RNA sequencing data were analyzed following a standard bioinformatics pipeline,^102^ as described in the Supporting Information. Briefly, FASTQ files were generated by Novogene and converted to sorted binary alignment map (BAM) files. Gene counts were determined by *GenomicAlignments* (R and Rstudio version 4.2.2) using the UCSC mm10 mouse as a reference. Sample variance for each tissue was assessed through a principal component analysis (PCA) (Figure S13–S15). Differential expression analysis was performed using a *DESeq2* pipeline (version 1.38.3)^103^ where DEGs were classified with false discovery rate (FDR) adjusted *p*-value <0.1 and log_2_ fold 0.3 for genes of interest to be considered significantly up- or downregulated in the striatum and the prefrontal cortex. In contrast, the liver DEGs were classified with adjusted *p*-value <0.1 and log_2_ fold 1 (Figures S6, S16, and S17). Because >30 DEGs were observed in the 6 mg/kg bw/d dose group in all the tissues investigated, pathway analyses were performed by iPathwayGuide (Advaita Corporation, Ann Arbor, Michigan).^42,43^ Furthermore, gene set analyses (GSA) were performed across all three exposure groups with *clusterProfiler*.^44^ In addition, we conducted a deconvolution process to explore PCB-mediated changes in cell populations of the striatum and prefrontal cortex based on single-cell RNA sequencing reference data (Figures S1 and S2).^104^

### Metabolomic Analysis of Striatum Samples

Metabolomic analyses were performed by the Northwest Metabolomics Research Center following published protocols (University of Washington, Seattle, Washington).^105,106^ Quality control samples included a pooled human plasma and pooled striatum extract. These samples were analyzed concurrently with the striatum samples to monitor instrument stability. Relative data of 361 metabolites reported for the striatum samples were analyzed using MetaboAnalyst 5.0.^107^ Relative values were filtered by interquartile range (>10%), normalized by sum, and log-transformed before further analysis. Data variability and group clustering are visualized through score plots in PCA, as shown in Figure S5.

### Multiomics Network Analysis

An interaction network analysis was performed to explore correlations between the PCB and OH-PCB levels and transcriptome. Paired analyses were conducted to determine the correlation between the brain PCB and OH-PCB levels and DEGs, expressed as transcripts per million (TPM)-normalized gene counts, in the striatum or prefrontal cortex. This analysis was performed using the partial least-squares (PLS) regression analysis and eigenvector centrality implemented by xMWAS (version 0.552).^83^ An analogous network analysis was performed with the liver PCB levels and transcriptome. Associations with absolute correlation coefficients above a threshold (>0.75 for the striatum, >0.7 for the prefrontal cortex, and >0.85 for the liver) and *P* < 0.05 were visualized and annotated using Cytoscape (version 3.10.1).^108^

### Statistical Analysis

The levels of PCBs and metabolites, adjusted for tissue wet weight, are expressed as the mean ± standard deviation. Significant differences in PCB and OH-PCB levels (data were log-transformed to ensure equal variance) by dose and tissue were assessed using 2-way ANOVA, followed by Tukey post hoc analysis, with *p* < 0.05 considered significantly different (Tables S2 and S3). Similarity coefficients cos θ were calculated with the formula:
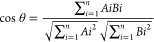
where *Ai* and *Bi* are the *i*th components of vectors *A* and *B*, respectively.^101^ Heatmaps of PCB and OH-PCB metabolite levels were generated with log-transformed values by GraphPad Prism (RRID:SCR_002798) version 10.0.2. Normalized gene counts are shown as violin plots representing the frequency distribution, with median and quartiles indicated by dotted lines. The adjusted *p*-values shown in the violin plots were determined using the *DESeq2* pipeline, as described above. Adjusted *p*-value <0.1 were considered significantly different from controls for all other analyses of the RNaseq data. All statistical analyses for RNA sequencing were conducted by R and Rstudio version 4.2.2. Interaction network analyses of brain PCB and OH-PCB levels and the brain transcriptome were performed with xMWAS (version 0.552)^83^ using a threshold of absolute correlation coefficients >0.7 for the prefrontal cortex, >0.75 for the striatum, and *p* < 0.05.

## Supporting Information Available

Description of chemicals, quality assurance/quality control (QA/QC), and RNA sequencing analysis; levels of PCBs and their metabolites; statistical analysis of PCB and OH-PCB levels by dose and tissue using 2-way ANOVA; abbreviations and unique identifiers; GC-MS/MS parameters; QA/QC results; bulk RNaseq deconvolution to single cell type estimates on the prefrontal cortex and striatum; plots of normalized gene counts for *Mapt*, drug-metabolizing enzymes, and genes altered in all PCB exposure groups compared to controls; PCA of striatal metabolomics data; Venn diagram of common DEGs in the prefrontal cortex, striatum, and liver of the high-dose exposure group and the liver from all exposure groups; normalized gene count plots of selected DEGs in the liver; network analysis of liver transcriptome and liver PCB and OH-PCB levels; PCA and volcano plots from pairwise comparisons of each treatment of MARBLES mixture versus the vehicle group; mass profile of the MARBLES mix; and animal bodyweights (PDF)

## Abbreviations

Abcc3: ATP-binding cassette subfamily C member 3
AhR: aryl hydrocarbon receptor
Arrb2: arrestin β 2
ATP5B: ATP synthase, subunit β
Atrx: transcriptional regulator ATRX
BAM: binary alignment map
Bdp1: transcription factor Bdp1
CAR: constitutive androstane receptor
Ces2a: carboxylesterase 2a
Ciart: circadian associated repressor of transcription
CYP: cytochrome P450
Dbp: D-site albumin promoter binding protein
DEG: differentially expressed gene
Derl3: derlin 3
Drd2: dopamine receptor D2
ER: endoplasmic reticulum
Etv4: ETS variant transcription factor 4
FDR: false discovery rate
Fos: Fos proto-oncogene, AP-1 transcription factor subunit
GC-MS/MS: gas chromatograph with tandem mass spectrometry
GSA: gene set analyses
H2–K2: histocompatibility 2, K region locus 2
KEGG: Kyoto encyclopedia of genes and genomes
Mafg: MAF BZIP transcription factor G
MAPK: mitogen-activated protein kinase
MARBLES: markers of autism risk in babies – learning early signs
MHC: major histocompatibility complex
MRP3: multidrug resistance-associated protein 3
Naga: α-*N*-acetylgalactosaminidase
Ncapd2: non-SMC condensin I complex subunit D2
Ngef: neuronal guanine nucleotide exchange factor
OH-PCB: hydroxylated polychlorinated biphenyl
Oprm1: opioid receptor mu 1
P7: postnatal day 7
P25: postnatal day 25
P30: postnatal day 30
P27: postnatal day 27
P32: postnatal day 32
Pars2: prolyl-tRNA synthetase 2
PBS: phosphate-buffered saline
PCA: principal component analysis
PCB: polychlorinated biphenyl
Pdgfb: platelet-derived growth factor subunit B
Per3: period circadian regulator 3
PLS: partial least-squares
PND: postnatal day
PPE: personal protective equipment
RIN: RNA integrity number
RyR: ryanodine receptor
Sh3pxd2a: SH3 and PX domain-containing protein 2A
Sdc3: syndecan 3
Slc27a1: solute carrier family 27 member 1
Tet3: tet methylcytosine dioxygenase 3
TPM: transcripts per million
Trib1: tribbles pseudokinase 1
Uba6: ubiquitin like modifier activating enzyme 6
Usp2: ubiquitin specific peptidase
