# Supplementary material for: Interactions of Polychlorinated Biphenyls and Their Metabolites with the Brain and Liver Transcriptome of Female Mice
Source: ACS Chem Neurosci. 2024 Oct 11;15(21):3991–4009. doi: 10.1021/acschemneuro.4c00367 (PMC11587508; doi:10.1021/acschemneuro.4c00367)
Supplement: Supplementary file 1 — cn4c00367_si_001.pdf [file cn4c00367_si_001.pdf]

SUPPORTING INFORMATION

INTERACTIONS OF POLYCHLORINATED BIPHENYLS AND THEIR METABOLITES WITH THE BRAIN AND LIVER TRANSCRIPTOME OF FEMALE MICE

Amanda J. Bullert[1,2,†], Hui Wang[1,†], Anthony E. Valenzuela[3], Kari Neier[4], Rebecca J.Wilson[3], Jessie R. Badley[3], Janine M. LaSalle[4], Xin Hu[5], Pamela J. Lein[3], Hans-Joachim Lehmler[1,6]*

[1]Department of Occupational and Environmental Health, University of Iowa, Iowa City, IA 52242, USA; [2]Interdisciplinary Graduate Program in Neuroscience, University of Iowa, Iowa City, IA 52242, USA; [3]Department of Molecular Biosciences, University of California, Davis, CA 95616, USA; [4]Department of Medical Microbiology and Immunology, University of California, Davis, CA 95616, USA; [5]Gangarosa Department of Environmental Health, Emory University, Atlanta, GA 30329, USA; [6]Interdisciplinary Graduate Program in Human Toxicology, University of Iowa, Iowa City, IA 52242, USA

Corresponding Author:
Dr. Hans-Joachim Lehmler
The University of Iowa
Department of Occupational and Environmental Health
University of Iowa Research Park, #221 IREH
Iowa City, IA 52242-5000
Phone: (319) 335-4310
Fax: (319) 335-4290
Email: hans-joachim-lehmler@uiowa.edu

[†] Both authors contributed equally to this manuscript.

Table of Contents

Chemicals S4

Quality assurance and quality control (QA/QC) S5

RNA sequencing analysis S6

Differential expression analysis S7

Visualization S7

Pathway and gene set analysis S7

Table S1. PCB and OH-PCB concentration (ng/g fresh weight) in tissues and serum S9

Table S2. The p-values of comparing PCB or OH-PCB levels in different dosing group, but within one tissue, using two-way ANOVA analysis with Tukey's post hoc test for multiple comparisons. S12

Table S3. The p-values of PCB and OH-PCB levels in different tissues, but within same dosing group, using two-way ANOVA analysis with Tukey's post hoc test for multiple comparisons. S14

Table S4. Abbreviations and unique identifiers of the analytical OH-PCB standards used in this study S16

Table S5. Precursor ions, product ions, and collision energies for each analyte in the GC-MS/MS analysis S18

Table S6. Ongoing Precision Recovery (OPR) for selected PCBs and OH-PCBs in method blanks and tissue matrices S20

Table S7. Recoveries (%) of surrogate standards for PCB and OH-PCB in different biological matrices S21

Table S8. Method Detection Limits (MDLs) and Limits of Detection (LODs) for PCBs from MARBLES mix and their possible OH-PCB metabolites S22

Fig S1. Bulk RNAseq deconvolution to single cell type estimates in the prefrontal cortex of mice exposed to different doses of the MARBLES mix S24

Fig S2. Bulk RNAseq deconvolution to single cell type estimates in the striatum (STN) of mice exposed to different doses of the MARBLES mix S25

Fig S3. Normalized gene expression of microtubule-associated protein tau (*Mapt*) in the (A) prefrontal cortex (PFC), (B) striatum (STN), and (C) liver following oral exposure to 0, 0.1, 1, or 6 mg/kg body weight/day of the MARBLES mix S26

Fig S4. Normalized gene count plots for drug-metabolizing genes, including (A) *Cyp1a2*, (B) *Cyp2a5*, (C) *Cyp2b10*, (D) *Cyp2c50*, (E) *Cyp3a41a*, (F) *Cyp2S1*, (G) *Cyp4x1*, and (H) *Sult2a1*, in the prefrontal cortex (PFC), striatum (STN), and liver samples from mice orally exposed to 0, 0.1, 1, or 6 mg/kg body weight/day of the MARBLES mix, reveals differences by tissue type and PCB dose S27

Fig S5. Principal Component Analysis (PCA) of metabolomic data from striatum samples from female mice exposed to 0, 0.1, 1, and 6 mg/kg body weight/day of the MARBLES mix shows not separation by exposure group S29

Fig S6. Volcano plots showing pairwise comparisons of RNA sequencing data from each MARBLES mix dose group versus the vehicle group in the liver S30

Fig S7. Venn diagram of common differentially expressed genes across tissue samples collected from mice exposed to 6 mg/kg of the MARBLES mix S31

Fig S8. Venn diagram of common differentially expressed genes across exposure groups in the liver samples collected from mice exposed to 0.1, 1, and 6 mg/kg body weight/day of the MARBLES mix S32

Fig S9. Normalized gene count plots of the seven differentially expressed genes identified in the Venn diagram in **Fig S8**, including (A) D-site albumin promoter binding protein (*Dbp*), (B) MAF BZIP transcription factor G (*Mafg*), (C) Tribbles pseudokinase 1 (*Trib1*), (D) circadian associated repressor of transcription (*Ciart*), (E) ubiquitin specific peptidase (*Usp2*), (F) neuronal guanine nucleotide exchange factor (*Ngef*), and (G) derlin 3 (*Derl3*) S33

Fig S10. Network analysis of liver transcriptome and liver PCB and OH-PCB levels identifying four clusters (**panel A**). S35

Fig S11. Mass profile of the MARBLES mix, a PCB mixture containing 12 PCB congeners detected in serum collected during the third trimester of women enrolled in the MARBLES study S37

Fig S12. Pre- and post-exposure body weights of female mice exposed to different doses of the MARBLES mix S38

Fig S13. Principal component analysis (PCA) plot of RNA sequencing data from liver samples from mice exposed to 0, 0.1, 1, or 6 mg/kg body weight/day of the MARBLES mix S39

Fig S14. Principal component analysis (PCA) plot of RNA sequencing data from prefrontal cortex samples from mice exposed to 0, 0.1, 1, or 6 mg/kg body weight/day of the MARBLES mix S40

Fig S15. Principal component analysis (PCA) plot of RNA sequencing data from striatum samples from mice exposed to 0, 0.1, 1, or 6 mg/kg body weight/day of the MARBLES mix S41

Fig S16. Volcano plots showing pairwise comparisons of RNA sequencing data from each MARBLES mix dose group versus the vehicle group in the prefrontal cortex S42

Fig S17. Volcano plots showing pairwise comparisons of RNA sequencing data from each MARBLES mix dose group versus the vehicle group in the striatum S43

References S44

Chemicals. The shorthand PCB nomenclature used in the manuscript is based on the table of polychlorinated biphenyls (PCBs) published by the US EPA.[1] The nomenclature of OH-PCBs is an abbreviated version of the PCB metabolite nomenclature proposed by Maervoet and co-workers, where the first number indicates the position of the OH-group on the biphenyl moiety and the second number reflects the number of the corresponding PCB congener.[2] The unique identifiers, including SMILES structure for each PCB congener and MeO-PCB, were described elsewhere.[3, 4] The unique identifiers of the OH-PCB standards used in this study are listed in **Table S1** below.

A PCB calibration standard was prepared for standard mixtures containing all 209 PCB congeners (AccuStandard, New Haven, Connecticut, USA). Two MeO-PCB calibration standard solutions were used to analyze OH-PCBs (after methylation to MeO-PCBs with diazomethane). The first MeO-PCB standard solution contained seventy-two methoxylated PCBs (MeO-PCBs) (70 mono-MeO-PCBs and two di-MeO-PCBs) provided by AccuStandard or Wellington Laboratories (Guelph, Ontario, Canada).[5] The second MeO-PCB standard solution consisted of fifty-two MeO-PCBs (28 mono-MeO-PCBs and 24 di-MeO-PCBs as standard Solution 2). These MeO-PCBs were synthesized through a Suzuki coupling reaction between a suitable benzene boronic acid and a methoxylated bromochlorobenzene,[6, 7] and authenticated as described.[8-14]

In addition, OH-PCB standards were synthesized and authenticated as described,[7, 11, 15-17] including 3,3'-dichlorobiphenyl-4-ol (4-OH-PCB11), 2,3',4-trichlorobiphenyl-4'-ol (4'-OH-PCB25), 2,2',5,5'-tetrachlorobiphenyl-4-ol (4-OH-PCB52), 2,2',3,5',6-pentachlorobiphenyl-4-ol (4-OH-PCB95), 2,2',3,5',6-pentachlorobiphenyl-5-ol (5-OH-PCB95), 2,2',4,4',5,5'-hexachloro-biphenyl-3-ol (3-OH-PCB153), and 2,2',3,4',5,5'-hexachlorobiphenyl-4-ol (4-OH-PCB146) in

methanol (100 ng/mL each). All other PCB and OH-PCB standards were purchased from AccuStandard (New Haven, Connecticut, USA).

4,4'-Dichlorobiphenyl (PCB15) and 2,3,4',5,6-pentachlorobiphenyl (PCB117) (100 ng/mL each in isooctane) were used as surrogate standards for PCBs. 2,5-Dichlorobiphenyl-4'-ol (4'-9), 2,2',3,4',6-pentachlorobiphenyl-4-ol (4-OH-PCB91), and 2,3,3',4,5,5'-hexachlorobiphenyl-4'-ol (4'-OH-PCB159) (100 ng/mL each in methanol) were the surrogate standards used for OH-PCBs. In addition, deuterium-labeled 2,4,6-trichlorobiphenyl-2',3',4',5',6'-d_5 (d-PCB30; CDN Isotopes, Quebec, Canada) and 2,2',3,4,4',5,6,6'-octachlorobiphenyl (PCB204) were used as a volume corrector.

All solvents (pesticide grade), sulfuric acid (concentrated), hydrochloric acid (concentrated), potassium chloride, sodium chloride, silica gel, and sodium sulfite were provided by Thermo Fisher Scientific (Fair Lawn, New Jersey, USA). Tetrabutylammonium (TBA) hydrogen sulfate was obtained from J.T. Baker (Phillipsburg, New Jersey, USA). As previously described, a solution of diazomethane for the derivatization of OH-PCBs was prepared using an Aldrich Mini Diazald® apparatus.[18]

Quality assurance and quality control (QA/QC). Solvent blanks, method blanks, and ongoing precision recovery (OPR) standard samples were analyzed in parallel with all experimental samples. Surrogate standards were added to each sample, and the analyte mass was corrected based on the corresponding surrogate standard recovery. The following formula was used to calculate the Method detection limit (MDL):

$$MDL = mean_{blank} + t_{0.01,\, n-1} * SD_{blank}$$

where $mean_{blank}$ is the mean of method blanks, $t_{0.01,\, n-1}$ is the Student's t-value for n – 1 degrees of freedom at the 99% confidence level, and SD_{blank} is the standard deviation of the method blanks. Similarly, the limits of detection (LODs) were calculated with the formula:

$$LOD = mean_{control} + t_{0.01,\, n-1} * SD_{control}$$

where $mean_{control}$ is the mean of control tissue measures, $t_{0.01,\, n-1}$ is the Student's t-value for n – 1 degrees of freedom at the 99% confidence level, and $SD_{control}$ is the standard deviation of the control tissue measures.

RNA sequencing and analysis. The striatum and prefrontal cortex were isolated and, together with the liver, prepared for RNA sequencing. Briefly, total RNA was extracted using a QIAGEN RNeasy Mini kit (QIAGEN, Germantown, Maryland). Samples were homogenized in a microcentrifuge tube with a Fisherbrand Pellet Pestle homogenizer. RNA purity and quantity were assessed with a NanoDrop and Qubit, respectively. A total of 63 samples had RNA integrity values ≥8.0 and were submitted to Novogene (Davis, California, USA) for Illumina RNA sequencing. Novogene performed an additional quality control step using an Agilent 5400 Fragment Analyzer System, followed by cDNA library construction using ribosomal depletion methods. Reads were sequenced to a depth of 20 million reads in 120 base pairs paired-end sequences per the Illumina 1.9 encoding Novogene protocols.

Data were processed in triplicate FASTQ files, which were demultiplexed and concatenated for each sample. Raw RNA-seq FASTQ files were generated by Novogene and further processed for differential gene expression. The FASTQ file quality was assessed with FASTQC (version 0.11.9). One FASTQ file from the liver in the 0.1 mg/kg bw/d exposure group was of poor quality and was removed from further analyses. Sequence reads were then mapped to the UCSC mm10 mouse reference genome using HISAT2 (version 2.1.0-4). The forward and

reverse read sequences from all files had a mapping train of >80% and were included in further analyses. Once sequencing alignment map (SAM) files were generated, they were immediately converted to sorted binary alignment map (BAM) files using SAMtools. Gene counts were determined by *GenomicAlignments* (R and Rstudio version 4.2.2) using the UCSC mm10 mouse gene transfer format (GTF) as a reference. The counting mode selected was "Union" to handle gaps and overlaps in sequences.

Differential expression analysis. Differential expression analysis was performed using a DESeq2 pipeline (version 1.38.3).[19] Count tables are prefiltered to remove low-count genes (< or = 10). Differentially expressed genes were defined as false discovery rate (FDR) Benjamini-Hochberg adjusted *p*-value <0.1 in the PCB exposed groups compared to the vehicle-exposed group for each tissue, while $\log_2$ fold change was set to a threshold of 0.3 for genes of interest to be considered significant up- or downregulated in the brain samples (striatum and prefrontal cortex). The thresholds to define a differentially expressed gene for the liver were an adjusted *p*-value <0.1 and a $\log_2$ fold change of 1.0 for genes.

Visualization. Data quality was assessed through the sample variation distance by generated principal component analysis (PCA) plots using the first two principal components (**Figs S12-14**). Volcano plots were generated based on $\log_2$ fold change and adjusted p-values to identify genes that exceeded the corresponding thresholds for differentially expressed genes for each tissue using *EnhancedVolcano* (**Figs S6, S15 &S16**). For genes of interest, normalized counts were plotted with individual points representing samples and the mean as a horizontal bar.

Pathway and gene set analysis. Additional analyses, such as pathway and gene set analysis (GSA), were conducted for further data interpretation. Pathway analysis, as implemented by iPathwayGuide (Advaita Corporation, Ann Arbor, Michigan, USA), allows the identification

of potential pathways contributing to the phenotypes based on the exposure group but requires that enough genes (>30) reach an adjusted p-value <0.1 from the DESeq2 analysis.[20] For each tissue, samples from the 6 mg/kg bw/d dose group compared to the vehicle control group met this threshold and were analyzed with iPathwayGuide to identify biological processes affected by PCB exposure.

Gene set analysis (GSA), in which associations of a collection of genes with specific biological processes (and not only differentially expressed genes) are included in a univariate functional class score, was performed for all exposure groups with *clusterProfiler*.[21, 22] Venn diagrams were generated with the shiny app associated with *VennDetail* to compare the overlap of genes with adjusted p-values <0.1 across exposure groups. Finally, for exploratory purposes, we conducted a deconvolution process based on single-cell RNA sequencing reference data to determine whether the cell populations of the striatum and PFC were affected by PCB exposure. This analysis was conducted via *MuSiC2* (**Fig S1 & S2**).[23]

Table S1. PCB and OH-PCB concentration (ng/g fresh weight) in tissues and serum. Values are mean ± SD.

PCB/OH-PCB	Serum			Liver			Brain		
	Low (n=5)	Medium (n=5)	High (n=6)	Low (n=5)	Medium (n=6)	High (n=6)	Low (n=5)	Medium (n=6)	High (n=6)
PCB 11	ND	0.323±0.723	0.627±0.974	0.354±0.793	1.65±0.92	7.08±2.3	ND	ND	ND
2-11	0.013 ±0.013	0.006 ±0.014	0.011 ±0.015	ND	ND	0.005 ±0.012	ND	ND	ND
4-11	0.058 ±0.041	1.23 ±0.54	5.49 ±2.76	0.453 ±0.120	1.50 ±0.50	7.92 ±3.80	ND	ND	ND
5-11	ND	ND	0.298 ±0.130	ND	ND	0.416 ±0.215	ND	0.001 ±0.001	0.001 ±0.001
6-11	ND	ND	ND	ND	ND	ND	ND	ND	ND
5,6-11	ND	ND	ND	ND	0.003 ±0.007	ND	ND	ND	0.003 ±0.006
2,5-11	ND	ND	ND	ND	ND	ND	ND	ND	ND
4,5-11	0.001 ±0.002	ND	0.034 ±0.04	ND	0.007 ±0.017	0.070 ±0.158	ND	ND	ND
PCB 28	19.5±2.1	190±46	1020±160	460±82	2570±845	11900 ±2930	40.5±7.2	392±99	1780±160
2'-28	0.065 ±0.013	0.437 ±0.084	1.54±0.48	0.024 ±0.014	0.112 ±0.072	0.449 ±0.202	ND	0.003 ±0.005	0.011 ±0.009
3-28	0.689 ±0.019	6.36 ±2.18	21.7±6.3	2.52±1.61	15.2±9.2	49.2±26.5	0.117 ±0.015	1.11 ±0.24	2.97 ±1.19
3'-28	5.68 ±1.21	70.1±32.2	318±143	136±67	1050 ±570	3780 ±1920	ND	0.356 ±0.107	1.31±0.56
5-28	1.57 ±0.39	15.8±8.0	81.1±41.2	79.0±40.2	549±272	2380 ±1370	ND	0.125 ±0.071	0.697 ±0.282
4'-25	ND	0.674 ±0.621	2.96±1.89	0.114 ±0.087	2.31±1.45	9.82±3.15	ND	ND	ND
PCB 52	ND	28.9±8.7	61.9±12.0	13.5±3.4	132±57	594±273	2.49±0.34	23.5±6.9	70.3±13.3
4-52	15.7±4.2	142±31	328±86	2.47±0.25	20.9±4.3	130±44	ND	0.576	1.69±1.10

PCB/OH-PCB	Serum			Liver			Brain		
	Low (n=5)	Medium (n=5)	High (n=6)	Low (n=5)	Medium (n=6)	High (n=6)	Low (n=5)	Medium (n=6)	High (n=6)
								±0.286	
4,4'-52	0.146±0.03	0.890±0.183	0.604±0.128	ND	ND	0.101±0.079	ND	ND	ND
PCB 84	ND	0.453 ±1.012	ND	ND	0.376 ±0.159	0.992 ±0.420	ND	ND	0.291 ±0.052
PCB 95	ND	3.69±8.25	6.51±7.16	1.64±0.86	10.7±5.5	34.8±16.4	2.56±4.09	4.18±2.96	7.15±1.96
4-95	4.59 ±1.20	28.6±9.9	37.2±10.8	0.229 ±0.094	1.31±0.38	5.00±1.94	ND	ND	ND
4'-95	0.049 ±0.024	0.368 ±0.198	0.395 ±0.166	0.167 ±0.085	0.970 ±0.423	3.52±2.04	ND	ND	ND
5-95	0.002 ±0.003	0.523 ±0.369	1.56±1.07	0.153 ±0.030	1.32±0.75	4.93±2.26	ND	ND	ND
4,5-95	0.083 ±0.025	0.430 ±0.119	1.07±0.46	0.045 ±0.035	0.245 ±0.133	1.86±1.74	0.004±0.007	0.006 ±0.008	0.004 ±0.005
3-103[c]	0.035 ±0.01	0.204 ±0.059	0.123 ±0.045	0.049 ±0.027	0.226 ±0.112	0.406 ±0.308	ND	ND	ND
PCB 101	ND	14.8±10.6	27.7±5.3	8.69±2.93	83.1±38.9	232±113	1.62±0.36	15.1±4.6	32.9±6.1
4'-101	0.515 ±0.137	5.15±1.85	9.90±3.25	0.321 ±0.185	2.74±1.57	12.2±9.1	ND	ND	0.066±0.082
6'-101	ND	ND	0.001 ±0.002	ND	ND	0.001 ±0.002	0.001 ±0.001	0.002 ±0.005	0.002 ±0.004
PCB 118	4.58 ±2.63	34.9±7.3	181±43	42.2±6.9	393±149	2760 ±1150	5.68±1.97	50.7±10.8	250±68
3-118	0.467 ±0.120	3.80±0.48	34.4±10.0	2.99±1.68	15.9±14.0	112±67	0.062 ±0.058	0.995±0.278	6.44±4.22
PCB 135	ND	0.526 ±1.176	1.01±1.10	1.04±0.54	6.61±3.85	8.14±3.13	0.052 ±0.117	1.42±0.44	2.11±0.34
PCB 138	3.95 ±0.99	13.6±3.4	51.8±15.6	49.0±13.7	141±47	711±285	2.02±0.38	15.1±3.4	70.7±18.0
3'-138	0.061	0.444	4.88±0.78	0.208	1.30±0.96	14.6±6.0	ND	0.044	0.568

PCB/OH-PCB	Serum			Liver			Brain		
	Low (n=5)	Medium (n=5)	High (n=6)	Low (n=5)	Medium (n=6)	High (n=6)	Low (n=5)	Medium (n=6)	High (n=6)
	±0.011	±0.132		±0.103				±0.028	±0.332
5-138	0.070 ±0.029	0.193 ±0.062	0.186 ±0.051	0.034 ±0.020	0.159 ±0.080	0.462 ±0.279	ND	ND	ND
PCB 149	ND	1.29±2.89	3.06±2.39	1.92±0.91	13.9±8.3	22.3±9.8	ND	3.24±0.97	5.28±0.82
PCB 153	4.19 ±1.11	25.4±5.5	117±36	28.6±6.7	222±88	1510±610	4.20±0.87	35.6±8.6	167±38
3-153	1.13 ±0.23	6.16±1.55	53.2±12.2	0.518 ±0.084	2.50±0.71	41.0±8.3	ND	0.058 ±0.090	1.37±0.32
4-146[c]	0.274 ±0.039	0.828 ±0.210	2.30±0.54	NA	0.030 ±0.034	0.171 ±0.183	ND	ND	ND
PCB 180	6.11 ±1.64	29.0±7.3	128±31	77.6±23.5	288±106	1730±660	4.42±0.59	39.0±8.8	181±29
3'-180	0.105 ±0.011	0.503 ±0.124	2.90±0.55	0.112 ±0.159	0.511 ±0.178	6.22±2.80	ND	ND	ND

Table S2. The p-values of comparing PCB or OH-PCB levels in different dosing group, but within one tissue, using two-way ANOVA analysis with Tukey's post hoc test for multiple comparisons.

PCB or OH-PCB	Brain			Liver			Serum		
	L vs M	L vs H	M vs H	L vs M	L vs H	M vs H	L vs M	L vs H	M vs H
PCB 11	NA	NA	NA	0.0037	<0.0001	<0.0001	NA	NA	0.8768
PCB 28	<0.0001	<0.0001	<0.0001	<0.0001	<0.0001	<0.0001	<0.0001	<0.0001	<0.0001
PCB 52	<0.0001	<0.0001	<0.0001	<0.0001	<0.0001	<0.0001	NA	NA	0.0569
PCB 84	0.9753	0.6435	0.7541	NA	NA	0.1867	NA	NA	0.7504
PCB 95	0.0132	<0.0001	0.0616	<0.0001	<0.0001	<0.0001	NA	NA	0.0716
PCB 101	<0.0001	<0.0001	0.0067	<0.0001	<0.0001	<0.0001	NA	NA	<0.0001
PCB 118	<0.0001	<0.0001	<0.0001	<0.0001	<0.0001	<0.0001	<0.0001	<0.0001	<0.0001
PCB 135	0.0052	0.0002	0.5540	<0.0001	<0.0001	0.4796	NA	NA	0.6448
PCB 138	<0.0001	<0.0001	<0.0001	<0.0001	<0.0001	<0.0001	0.0057	<0.0001	0.0004
PCB 149	NA	NA	0.2269	<0.0001	<0.0001	0.0416	NA	NA	0.0629
PCB 153	<0.0001	<0.0001	<0.0001	<0.0001	<0.0001	<0.0001	<0.0001	<0.0001	<0.0001
PCB 180	<0.0001	<0.0001	<0.0001	<0.0001	<0.0001	<0.0001	<0.0001	<0.0001	<0.0001
4-11	NA	NA	NA	0.0302	<0.0001	<0.0001	<0.0001	<0.0001	<0.0001
5-11	>0.9999	>0.9999	>0.9999	NA	NA	NA	NA	NA	NA
4,5-11	NA	NA	NA	NA	NA	NA	NA	NA	NA
2'-28	NA	NA	0.9943	0.9203	0.2313	0.3903	0.0719	<0.0001	<0.0001
3-28	<0.0001	<0.0001	<0.0001	<0.0001	<0.0001	<0.0001	<0.0001	<0.0001	<0.0001
3'-28	NA	NA	<0.0001	<0.0001	<0.0001	<0.0001	<0.0001	<0.0001	<0.0001
5-28	NA	NA	<0.0001	<0.0001	<0.0001	<0.0001	<0.0001	<0.0001	<0.0001
4'-25	NA	NA	NA	<0.0001	<0.0001	<0.0001	NA	NA	<0.0001
4-52	NA	NA	<0.0001	<0.0001	<0.0001	<0.0001	<0.0001	<0.0001	<0.0001
4,4'-52	NA	NA	NA	NA	NA	NA	0.0008	0.0281	0.4201
4'-95	NA	NA	NA	0.0406	<0.0001	0.0005	0.1395	0.0798	0.9837
4-95	NA	NA	NA	0.0079	<0.0001	<0.0001	<0.0001	<0.0001	0.0788
5-95	NA	NA	NA	0.0048	<0.0001	<0.0001	0.0104	<0.0001	0.0006
4,5-95	0.9998	>0.9999	0.9997	0.6878	0.0001	0.0014	0.1059	<0.0001	0.0175
4'-101	NA	NA	NA	<0.0001	<0.0001	<0.0001	<0.0001	<0.0001	<0.0001
3-103	NA	NA	NA	0.7422	0.3882	0.8167	0.5093	0.8098	0.8520

PCB or OH-PCB	Brain			Liver			Serum		
	**L vs M**	**L vs H**	**M vs H**	**L vs M**	**L vs H**	**M vs H**	**L vs M**	**L vs H**	**M vs H**
3-118	<0.0001	<0.0001	<0.0001	<0.0001	<0.0001	<0.0001	<0.0001	<0.0001	<0.0001
3'-138	NA	NA	<0.0001	0.0151	<0.0001	<0.0001	0.0637	<0.0001	<0.0001
5-138	NA	NA	NA	0.8500	0.2445	0.5056	0.7011	0.7081	0.9988
4-146	NA	NA	NA	NA	NA	0.8195	0.0240	<0.0001	<0.0001
3-153	NA	NA	<0.0001	0.0003	<0.0001	<0.0001	<0.0001	<0.0001	<0.0001
3'-180	NA	NA	NA	0.2956	<0.0001	<0.0001	0.0630	<0.0001	<0.0001

PCB and OH-PCB levels were log-transformed to ensure equal variance. Individual PCB and OH-PCB levels in different dosing groups but within one tissue were compared using two-way ANOVA and Tukey's post hoc test for multiple comparisons. P-values are not available (NA) when groups have all the values below LOD.

Table S3. The p-values of PCB and OH-PCB levels in different tissues, but within same dosing group, using two-way ANOVA analysis with Tukey's post hoc test for multiple comparisons.

PCB or OH-PCB	Low			Medium			High		
	Brain vs Liver	Brain vs Serum	Liver vs Serum	Brain vs Liver	Brain vs Serum	Liver vs Serum	Brain vs Liver	Brain vs Serum	Liver vs Serum
PCB 11	NA	NA	NA	NA	NA	0.0555	NA	NA	<0.0001
PCB 28	<0.0001	0.009	<0.0001	<0.0001	0.0525	<0.0001	<0.0001	0.0752	<0.0001
PCB 52	<0.0001	NA	NA	<0.0001	0.7872	<0.0001	<0.0001	0.8762	<0.0001
PCB 84	NA	NA	NA	0.0455	0.0318	0.9656	0.1043	<0.0001	0.0244
PCB 95	0.7438	NA	NA	0.0100	0.0089	<0.0001	<0.0001	0.0094	<0.0001
PCB 101	<0.0001	NA	NA	<0.0001	0.3828	<0.0001	<0.0001	0.7831	<0.0001
PCB 118	<0.0001	0.3025	<0.0001	<0.0001	0.4626	<0.0001	<0.0001	0.4189	<0.0001
PCB 135	0.0182	NA	NA	0.0011	0.1149	<0.0001	0.0002	0.0628	<0.0001
PCB 138	<0.0001	0.0999	<0.0001	<0.0001	0.9384	<0.0001	<0.0001	0.4263	<0.0001
PCB 149	NA	NA	NA	0.0003	0.0031	<0.0001	<0.0001	0.0213	<0.0001
PCB 153	<0.0001	0.999	<0.0001	<0.0001	0.5464	<0.0001	<0.0001	0.3145	<0.0001
PCB 180	<0.0001	0.5278	<0.0001	<0.0001	0.595	<0.0001	<0.0001	0.3415	<0.0001
4-11	NA	NA	0.0025	NA	NA	0.6851	NA	NA	0.1760
5-11	>0.9999	NA	NA	>0.9999	NA	NA	0.1361	0.3123	0.8918
4,5-11	NA	NA	NA	NA	NA	NA	NA	NA	0.9877
2'-28	NA	NA	0.9079	0.7596	0.0461	0.2016	0.1129	<0.0001	0.0048
3-28	<0.0001	<0.0001	<0.0001	<0.0001	<0.0001	<0.0001	<0.0001	<0.0001	0.0001
3'-28	NA	NA	<0.0001	<0.0001	<0.0001	<0.0001	<0.0001	<0.0001	<0.0001
5-28	NA	NA	<0.0001	<0.0001	<0.0001	<0.0001	<0.0001	<0.0001	<0.0001
4'-25	NA	NA	NA	NA	NA	<0.0001	NA	NA	<0.0001
4-52	NA	NA	<0.0001	<0.0001	<0.0001	<0.0001	<0.0001	<0.0001	<0.0001
4,4'-52	NA	NA	NA	NA	NA	NA	NA	NA	0.0829
4'-95	NA	NA	0.5043	NA	NA	0.0508	NA	NA	<0.0001
4-95	NA	NA	<0.0001	NA	NA	<0.0001	NA	NA	<0.0001
5-95	NA	NA	0.2934	NA	NA	0.0216	NA	NA	<0.0001

PCB or OH-PCB	Low			Medium			High		
	Brain vs Liver	Brain vs Serum	Liver vs Serum	Brain vs Liver	Brain vs Serum	Liver vs Serum	Brain vs Liver	Brain vs Serum	Liver vs Serum
4,5-95	0.9085	0.7015	0.9213	0.3135	0.054	0.6185	<0.0001	0.0002	0.4802
4'-101	NA	NA	0.286	NA	NA	0.0009	<0.0001	<0.0001	0.9992
3-103	NA	NA	0.9892	NA	NA	0.9939	NA	NA	0.472
3-118	<0.0001	0.002	<0.0001	<0.0001	<0.0001	<0.0001	<0.0001	<0.0001	<0.0001
3'-138	NA	NA	0.3653	<0.0001	0.0827	0.0213	<0.0001	<0.0001	<0.0001
5-138	NA	NA	0.9311	NA	NA	0.9785	NA	NA	0.5025
4-146	NA	NA	NA	NA	NA	0.0005	NA	NA	<0.0001
3-153	NA	NA	0.0013	<0.0001	<0.0001	<0.0001	<0.0001	<0.0001	0.3227
3'-180	NA	NA	0.9999	NA	NA	0.9999	NA	NA	0.004

PCB and OH-PCB levels were log-transformed to ensure equal variance. Individual PCB and OH-PCB levels in different tissues but within the same dosing group were compared using two-way ANOVA and Tukey's post hoc test for multiple comparisons. P-values are not available (NA) when groups have all the values below LOD.

Table S4. Abbreviations and unique identifiers of the analytical OH-PCB standards used in this study.

Abbreviation	IUPAC Name	Formula	Isomeric SMILES	InChI	InChIKey	CAS Registry Number	CAS Registry URL	PubChem CID	PubChem Link	DTXSID	Comptox Link
4'-9	4-(2,5-dichlorophenyl)phenol	C12H8Cl2O	C1=CC(=CC=C1C2=C(C=CC(=C2)Cl)Cl)O	InChI=1S/C12H8Cl2O/c13-9-3-6-12(14)11(7-9)8-1-4-10(15)5-2-8/h1-7,15H	BTORSXCJJIWNIS-UHFFFAOYSA-N	53905-28-5	https://commonchemistry.cas.org/detail?cas_rn=53905-28-5	91653	https://pubchem.ncbi.nlm.nih.gov/compound/91653	DTXSID0022351	https://comptox.epa.gov/dashboard/chemical/details/DTXSID0022351
4-91	2,3,5-trichloro-4-(2,4-dichlorophenyl)phenol	C12H5Cl5O	C1=CC(=C(C(=C1Cl)Cl)C2=C(C=C(C(=C2Cl)Cl)O)Cl	InChI=1S/C12H5Cl5O/c13-5-1-2-6(7(14)3-5)10-8(15)4-9(18)11(16)12(10)17/h1-4,18H	RSKPYFJMALAIJJ-UHFFFAOYSA-N	NA	NA	101788493	https://pubchem.ncbi.nlm.nih.gov/compound/101788493	NA	NA
4'-159	2,6-dichloro-4-(2,3,4,5-tetrachlorophenyl)phenol	C12H4Cl6O	C1=C(C=C(C(=C1Cl)O)Cl)C2=CC(=C(C(=C2Cl)Cl)Cl)Cl	InChI=1S/C12H4Cl6O/c13-6-3-5(9(16)11(18)10(6)17)4-1-7(14)12(19)8(15)2-4/h1-3,19H	PZAKBNHYWBSZAF-UHFFFAOYSA-N	158076-63-2	https://commonchemistry.cas.org/detail?cas_rn=158076-63-2	178005	https://pubchem.ncbi.nlm.nih.gov/compound/178005	DTXSID70166369	https://comptox.epa.gov/dashboard/DTXSID70166369
4-11	2-chloro-4-(3-chlorophenyl)phenol	C12H8Cl2O	C1=CC(=CC(=C1)Cl)C2=CC(=C(C=C2)O)Cl	InChI=1S/C12H8Cl2O/c13-10-3-1-2-8(6-10)9-4-5-12(15)11(14)7-9/h1-7,15H	JOHAARQQFBMIOV-UHFFFAOYSA-N	53890-78-1	https://commonchemistry.cas.org/detail?cas_rn=53890-78-1	186674	https://pubchem.ncbi.nlm.nih.gov/compound/186674	DTXSID10202159	https://comptox.epa.gov/dashboard/DTXSID10202159
4'-25	2-chloro-4-(2,4-dichlorophenyl)phenol	C12H7Cl3O	C1=CC(=C(C=C1C2=C(C=C(C=C2)Cl)Cl)Cl)O	InChI=1S/C12H7Cl3O/c13-8-2-3-9(10(14)6-8)7-1-4-12(16)11(15)5-7/h1-6,16H	IPQDZKABLRZERH-UHFFFAOYSA-N	358767-68-7	https://commonchemistry.cas.org/detail?cas_rn=358767-68-7	53221454	https://pubchem.ncbi.nlm.nih.gov/compound/53221454	DTXSID50686095	https://comptox.epa.gov/dashboard/chemical/details/DTXSID50686095
4-52	2,5-dichloro-4-(2,5-dichlorophenyl)phenol	C12H6Cl4O	C1=CC(=C(C=C1Cl)C2=CC(=C(C=C2Cl)O)Cl)Cl	InChI=1S/C12H6Cl4O/c13-6-1-2-9(14)7(3-6)8-4-11(16)12(17)5-10(8)15/h1-5,17H	ZKDSNFDCQYBBIU-UHFFFAOYSA-N	51274-68-1	https://commonchemistry.cas.org/detail?cas_rn=51274-68-1	39971	https://pubchem.ncbi.nlm.nih.gov/compound/39971	DTXSID10199272	https://comptox.epa.gov/dashboard/DTXSID10199272

Abbreviation	IUPAC Name	Formula	Isomeric SMILES	InChI	InChIKey	CAS Registry Number	CAS Registry URL	PubChem CID	PubChem Link	DTXSID	Comptox Link
4-95	2,3,5-trichloro-4-(2,5-dichlorophenyl)phenol	C12H5Cl5O	C1=CC(=C(C=C1Cl)C2=C(C=C(C(=C2Cl)Cl)O)Cl)Cl	InChI=1S/C12H5Cl5O/c13-5-1-2-7(14)6(3-5)10-8(15)4-9(18)11(16)12(10)17/h1-4,18H	VLOXUAHEXUHYTO-UHFFFAOYSA-N	NA	NA	102344102	https://pubchem.ncbi.nlm.nih.gov/compound/102344102	NA	NA
5-95	2,4,5-trichloro-3-(2,5-dichlorophenyl)phenol	C12H5Cl5O	C1=CC(=C(C=C1Cl)C2=C(C(=CC(=C2Cl)Cl)O)Cl)Cl	InChI=1S/C12H5Cl5O/c13-5-1-2-7(14)6(3-5)10-11(16)8(15)4-9(18)12(10)17/h1-4,18H	NGZZCCQHHGJRSN-UHFFFAOYSA-N	NA	NA	102344104	https://pubchem.ncbi.nlm.nih.gov/compound/102344104	NA	NA
3-153	2,3,6-trichloro-5-(2,4,5-trichlorophenyl)phenol	C12H4Cl6O	C1=C(C(=CC(=C1Cl)Cl)Cl)C2=CC(=C(C(=C2Cl)O)Cl)Cl	InChI=1S/C12H4Cl6O/c13-6-3-8(15)7(14)1-4(6)5-2-9(16)11(18)12(19)10(5)17/h1-3,19H	ZVJPNYXNOYRCIJ-UHFFFAOYSA-N	54284-55-8	https://commonchemistry.cas.org/detail?cas_rn=54284-55-8	6452977	https://pubchem.ncbi.nlm.nih.gov/compound/6452977	DTXSID90202637	https://comptox.epa.gov/dashboard/DTXSID90202637
4-146	2,3,6-trichloro-4-(2,4,5-trichlorophenyl)phenol	C12H4Cl6O	C1=C(C(=CC(=C1Cl)Cl)Cl)C2=CC(=C(C(=C2Cl)Cl)O)Cl	InChI=1S/C12H4Cl6O/c13-6-3-8(15)7(14)1-4(6)5-2-9(16)12(19)11(18)10(5)17/h1-3,19H	KVRQWFNZIYFJRU-UHFFFAOYSA-N	145413-90-7	https://commonchemistry.cas.org/detail?cas_rn=145413-90-7	3050412	https://pubchem.ncbi.nlm.nih.gov/compound/3050412	DTXSID60163004	https://comptox.epa.gov/dashboard/DTXSID60163004

NA: not available.

Table S5. Precursor ions, product ions, and collision energies for each analyte in the GC-MS/MS analysis.

Analyte	Precursor Ion (*m/z*)	Product Ion (*m/z*)	Collision Energy (eV)
4'-9	252	209	20
PCB 11	222	152	25
2-11	252	202	25
4-11	252	209	25
5-11	252	222	20
6-11	252	202	25
5,6-11	282	232	25
2,5-11	282	232	25
4,5-11	282	204	25
PCB 15	222	152	25
PCB 28	256	186	25
d-PCB 30	261	191	30
2'-28	286	236	25
3-28	286	243	25
3'-28	286	243	25
5-28	286	243	25
4'-25	286	243	25
PCB 52	292	222	25
4-52	322	279	20
4,4'-52	352	337	20
PCB 84	326	256	25
4-91	356	313	25
PCB 95	326	256	25
4-95	356	313	25
4'-95	356	313	25
5-95	356	313	25
4,5-95	386	343	25
3-103	356	306	25
PCB 101	326	256	25
4'-101	356	313	25
6'-101	356	306	25
PCB 117	326	256	25
PCB 118	326	256	20
3-118	356	313	25
PCB 135	360	290	25
PCB 138	360	290	25
3'-138	390	347	25
5-138	390	347	25
PCB 149	360	290	25
PCB 153	360	290	25
3-153	390	347	25

Analyte	Precursor Ion (*m/z*)	Product Ion (*m/z*)	Collision Energy (eV)
4-146	390	347	25
4'-159	390	375	15
PCB 180	394	323	25
3'-180	424	381	25
PCB 204	430	358	25

Table S6. Ongoing Precision Recovery (OPR) for selected PCBs and OH-PCBs in method blanks and tissue matrices. Values are mean ± SD.

PCB/OH-PCB	Recovery in method blank (%, n = 6)	Recovery in serum (%, n = 7)	Recovery in liver (%, n = 5)	Recovery in brain (%, n = 4)
PCB 11	78 ± 6	88 ± 6	78 ± 10	74 ± 5
PCB 15	74 ± 5	86 ± 5	81 ± 19	58 ± 7
PCB 28	81 ± 5	91 ± 5	84 ± 6	80 ± 5
4'-9	86 ± 5	92 ± 5	76 ± 17	61 ± 8
PCB 52	82 ± 4	95 ± 5	82 ± 6	81 ± 5
4-11	87 ± 7	82 ± 7	80 ± 23	76 ± 3
PCB 95	85 ± 3	98 ± 4	83 ± 8	83 ± 4
PCB 84	85 ± 4	95 ± 3	82 ± 13	84 ± 4
4'-25	91 ± 5	95 ± 4	85 ± 17	81 ± 3
PCB 101	85 ± 3	101 ± 4	89 ± 3	84 ± 3
4-52	91 ± 2	98 ± 2	86 ± 13	87 ± 6
PCB 117	83 ± 4	97 ± 2	89 ± 4	87 ± 9
PCB 135	91 ± 5	98 ± 8	81 ± 12	86 ± 6
PCB 149	91 ± 5	98 ± 8	82 ± 11	84 ± 6
5-95	91 ± 2	96 ± 2	84 ± 18	84 ± 4
PCB 118	87 ± 5	98 ± 2	90 ± 4	84 ± 3
4-95	91 ± 2	96 ± 2	85 ± 17	83 ± 3
4-91	93 ± 2	99 ± 3	88 ± 14	83 ± 4
PCB 153	91 ± 5	98 ± 8	85 ± 5	86 ± 7
PCB 138	93 ± 5	99 ± 6	83 ± 8	85 ± 6
3-153	94 ± 5	96 ± 4	84 ± 9	70 ± 14
4-146	94 ± 7	97 ± 4	81 ± 8	81 ± 7
PCB 180	93 ± 2	97 ± 4	84 ± 4	86 ± 5
4'-159	102 ± 18	103 ± 12	80 ± 8	83 ± 4

Table S7. Recoveries (%) of surrogate standards for PCB and OH-PCB in different biological matrices. Values are mean ± SD.

**Surrogate Standards**	**Serum (n=30)**	**Liver (n=31)**	**Brain (n=24)**
PCB 15	78 ± 6	66 ± 10	64 ± 13
4'-9	88 ± 9	69 ± 12	64 ± 10
PCB 117	90 ± 9	88 ± 6	85 ± 16
4-91	98 ± 5	76 ± 14	88 ± 6
4'-159	97 ± 13	78 ± 6	85 ± 6

Table S8. Method Detection Limits (MDLs) and Limits of Detection (LODs) for PCBs from MARBLES mix and their possible OH-PCB metabolites.

PCB/OH-PCB	MDL[a] (ng) (n=18)	LOD[b] (ng/g)		
		Liver (n=7)	Serum (n=7)	Brain (n=4)
PCB 11	0.39	1.63	1.45	4.22
2-11	<0.01	0.02	<0.01	0.02
4-11	0.07	0.23	0.01	<0.01
5-11	0.05	0.27	<0.01	<0.01
6-11	0.13	0.55	2.11	2.71
5,6-11	<0.01	0.01	0.03	0.01
2,5-11	0.02	0.08	0.31	0.46
4,5-11	<0.01	0.01	<0.01	<0.01
PCB 28	0.10	14.95	0.99	3.42
2'-28	<0.01	<0.01	<0.01	<0.01
3-28	0.01	0.08	0.11	0.07
3'-28	0.01	8.32	0.04	0.11
5-28	<0.01	4.92	0.03	0.05
4'-25[c]	0.02	0.01	0.20	<0.01
PCB 52	0.94	0.73	10.52	0.93
4-52	0.14	0.23	0.45	0.34
4,4'-52	0.04	0.13	<0.01	0.65
PCB 84	0.15	0.11	1.54	0.24
PCB 95	1.00	0.47	10.72	0.88
4-95	<0.01	0.04	<0.01	<0.01
4'-95	<0.01	<0.01	<0.01	<0.01
5-95	<0.01	<0.01	<0.01	<0.01
4,5-95	<0.01	0.02	<0.01	<0.01
3-103[c]	<0.01	<0.01	<0.01	<0.01
PCB 101	1.10	0.47	11.38	0.72
4'-101	<0.01	0.01	0.02	<0.01
6'-101	<0.01	<0.01	<0.01	<0.01
PCB 118	0.39	1.44	4.40	1.28
3-118	0.11	1.04	0.21	0.02
PCB 135	0.12	0.04	1.84	0.22
PCB 138	0.34	2.36	2.19	0.35
3'-138	<0.01	<0.01	<0.01	<0.01
5-138	<0.01	<0.01	0.03	<0.01
PCB 149	0.52	0.18	3.93	0.88
PCB 153	0.36	1.15	2.45	0.73
3-153	<0.01	0.04	0.07	0.13
4-146[c]	0.01	0.03	0.20	0.33
PCB 180	0.02	2.77	0.03	<0.01
3'-180	<0.01	0.18	<0.01	3.08

[a] MDL, Method Detection Limits (ng) were calculated using the formula: MDL= $mean_{blank} + t_{0.01, n-1} * SD_{blank}$, where $mean_{blank}$ is the mean of method blanks, $t_{0.01, n-1}$ is Student's t-value for n – 1 degrees of freedom at the 99% confidence level, and SD_{blank} is the standard deviation of the method blanks. N = 14
[b] LOD, Limit of Detection (ng/g tissue weight) were adjusted by tissue mass and were calculated from formula: LOD= $mean_{control} + t_{0.01, n-1} * SD_{control}$, where $mean_{control}$ is the mean of control tissue measures, $t_{0.01, n-1}$ is Student's t-value for n – 1 degrees of freedom at the 99% confidence level, and $SD_{control}$ is the standard deviation of the control tissue measures.
[c] Possible metabolites due to NIH shift.

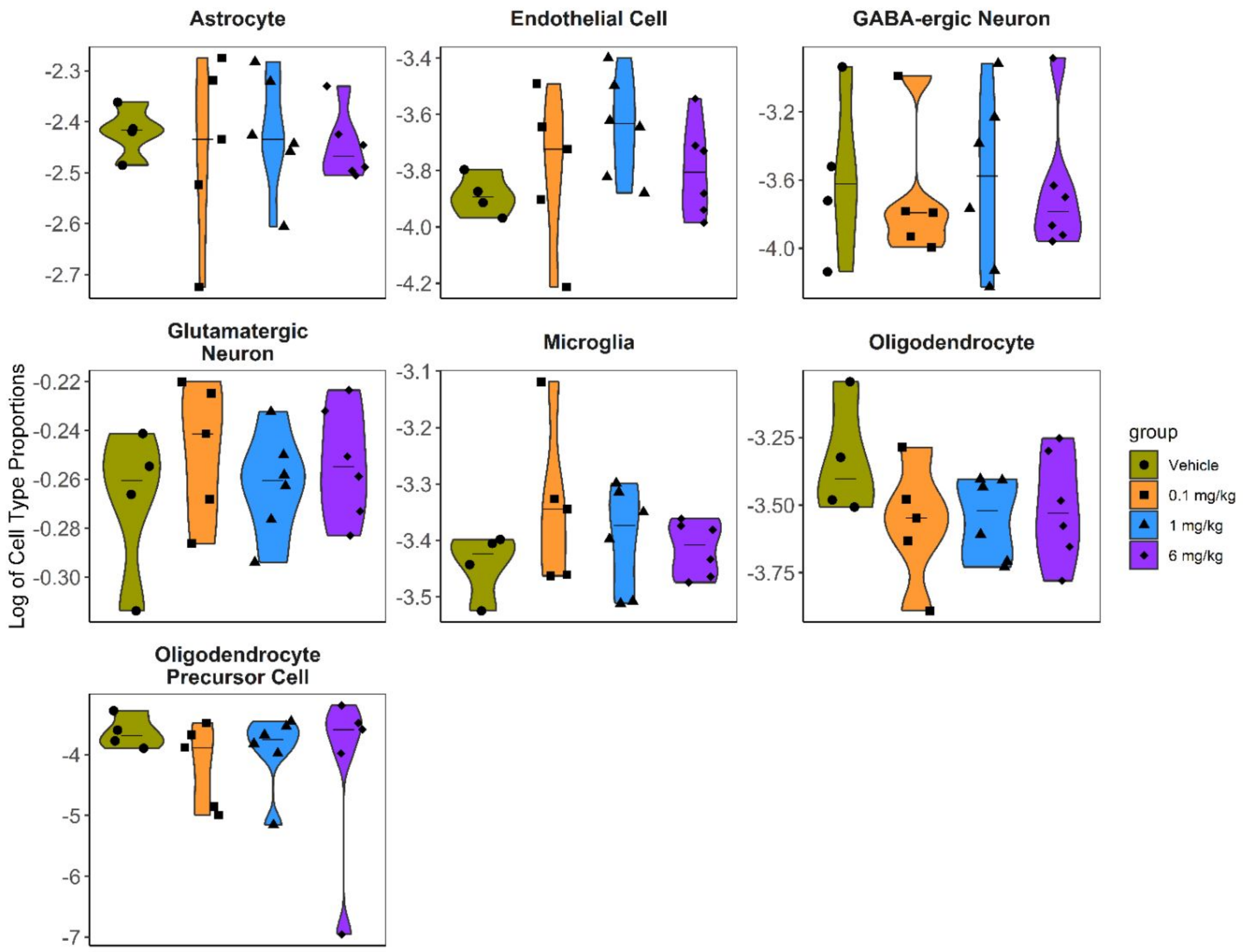

Fig S1. Bulk RNAseq deconvolution to single cell type estimates in the prefrontal cortex of mice exposed to different doses of the MARBLES mix. Violin plots represent the distribution of log cell type proportions compared to a single cell RNAseq experiment. Individual points indicate individual samples, and color identifies the exposure group.

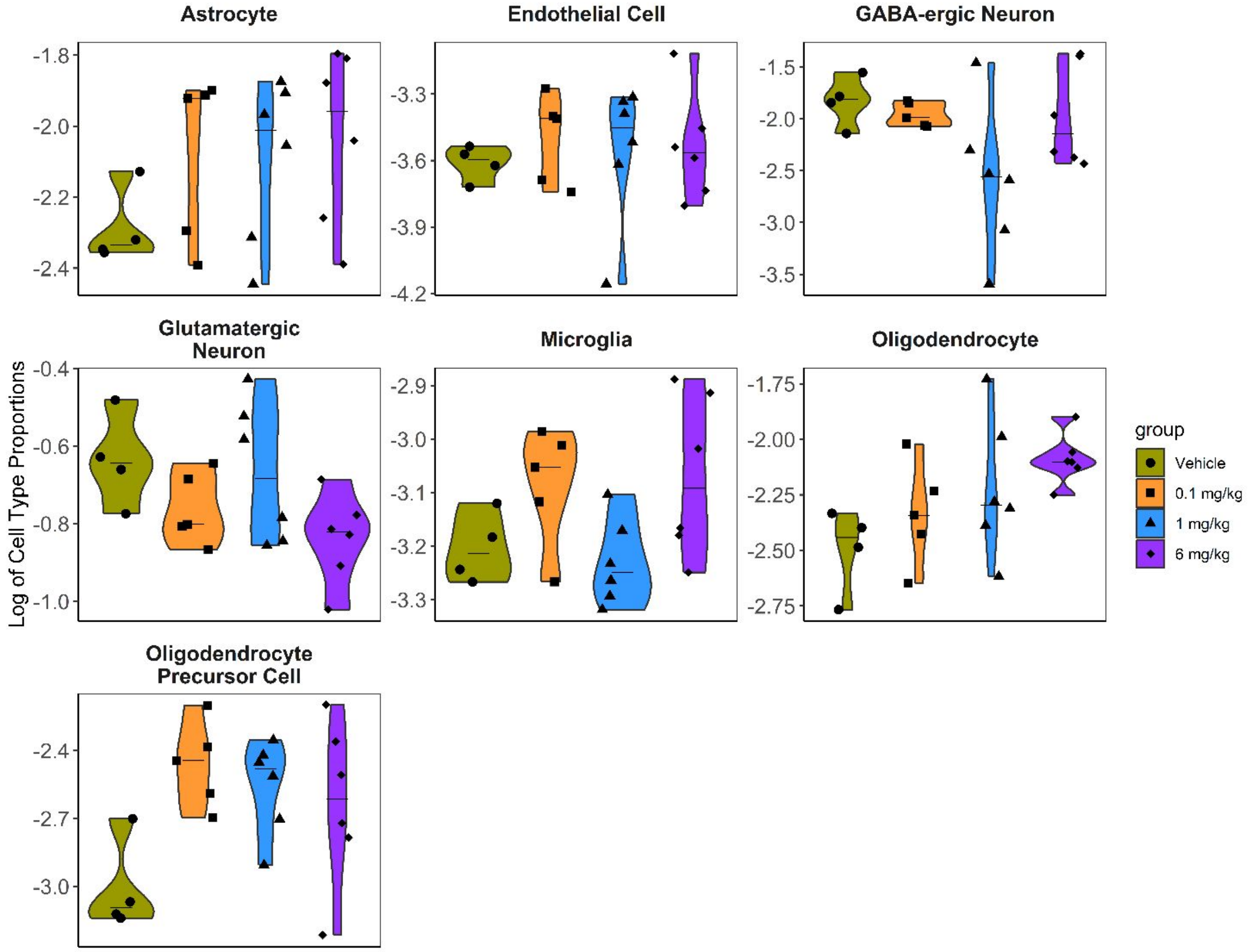

Fig S2. Bulk RNAseq deconvolution to single cell type estimates in the striatum (STN) of mice exposed to different doses of the MARBLES mix. Violin plots represent the distribution of log cell type proportions compared to a single cell RNAseq experiment. Individual points indicate individual samples, and color identifies the exposure group.

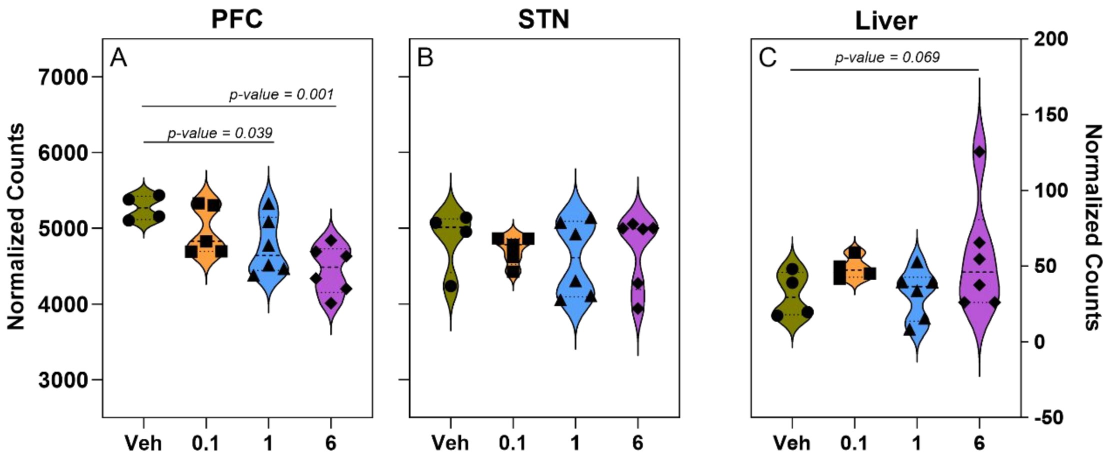

Fig S3. Normalized gene expression of microtubule-associated protein tau (*Mapt*) in the (A) prefrontal cortex (PFC), (B) striatum (STN), and (C) liver following oral exposure to 0, 0.1, 1, or 6 mg/kg body weight/day of the MARBLES mix. Violin plots are expressed as mean ± standard error with individual dots representing different animals (N = 4 to 6). For a description of the statistical analysis, see the manuscript.

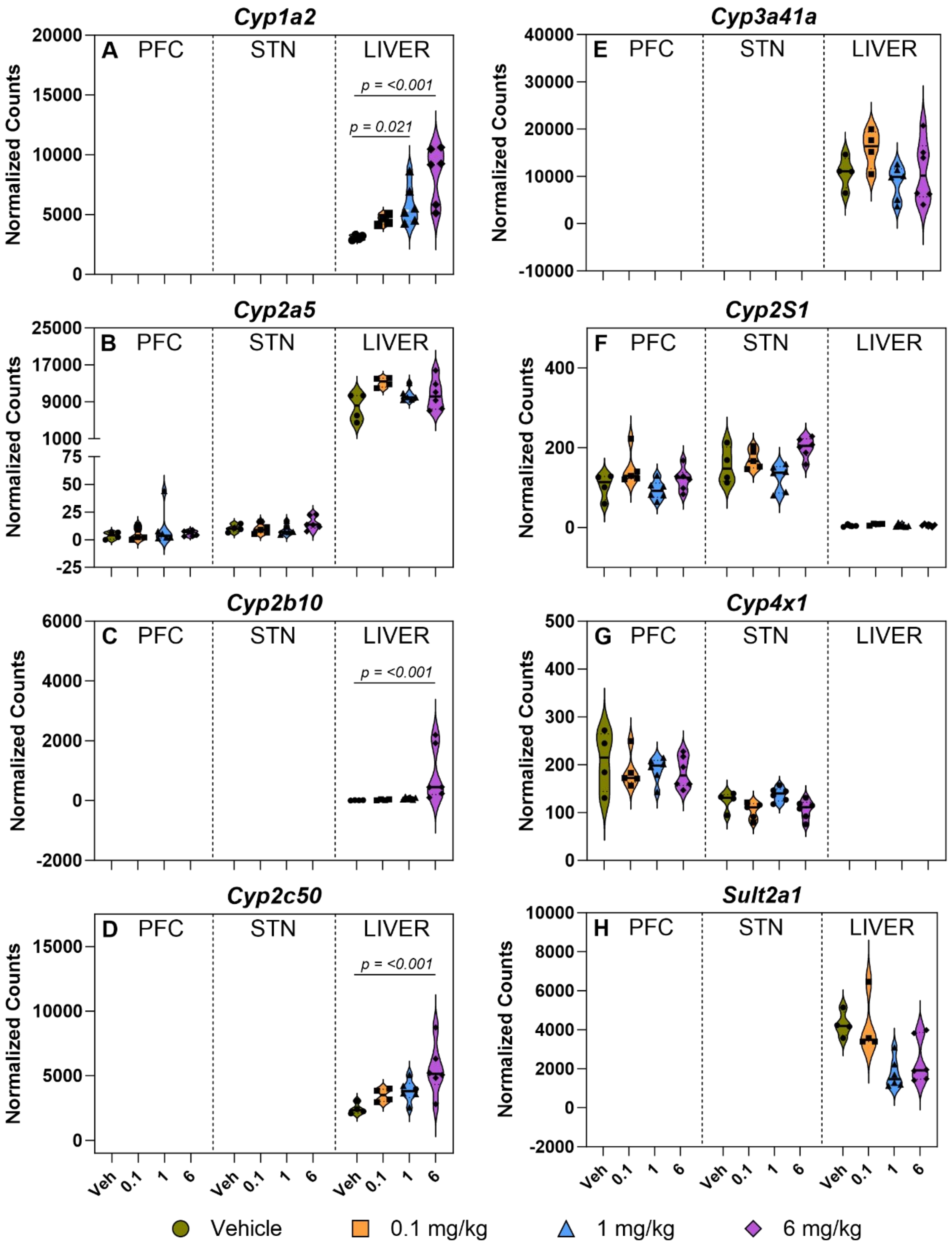

Fig S4. Normalized gene count plots for drug-metabolizing genes, including (A) *Cyp1a2*, (B) *Cyp2a5*, (C) *Cyp2b10*, (D) *Cyp2c50*, (E) *Cyp3a41a*, (F) *Cyp2S1*, (G) *Cyp4x1*, and (H) *Sult2a1*, in

the prefrontal cortex (PFC), striatum (STN), and liver samples from mice orally exposed to 0, 0.1, 1, or 6 mg/kg body weight/day of the MARBLES mix, reveals differences by tissue type and PCB dose. Violin plots are expressed as mean ± standard error with individual dots representing different animals. (N = 4 to 6). For a description of the statistical analysis, see the manuscript.

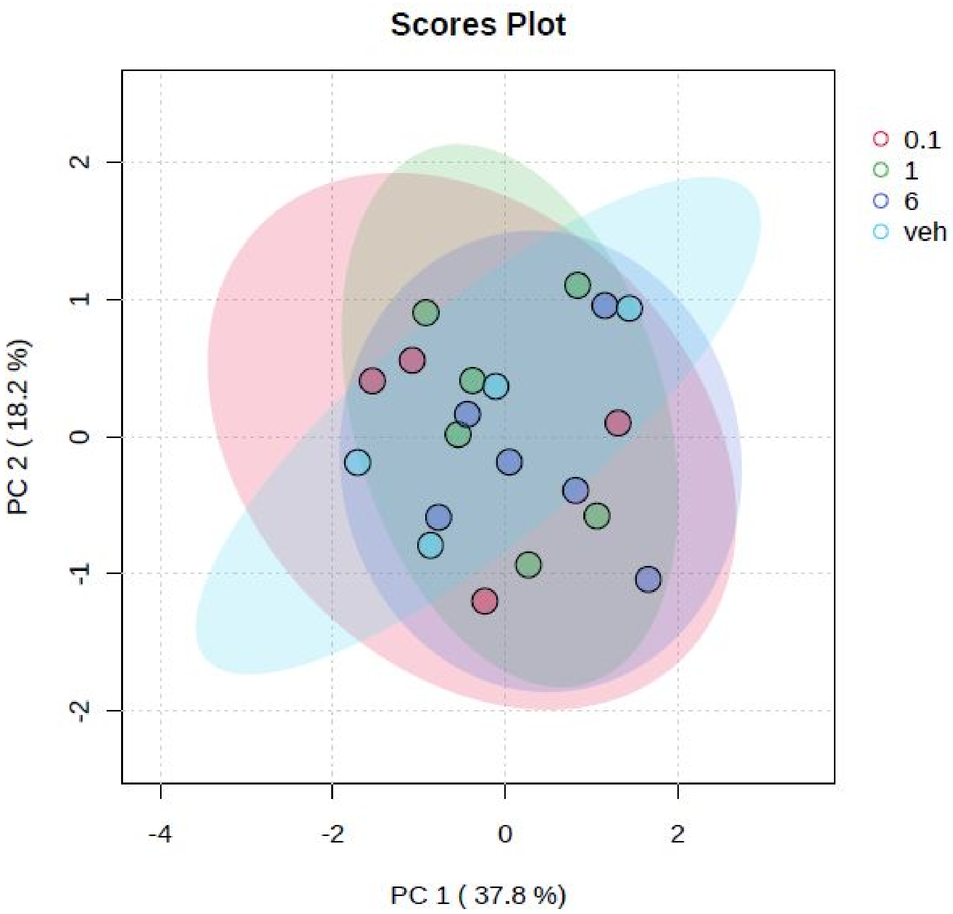

Fig S5. Principal Component Analysis (PCA) of metabolomic data from striatum samples from female mice exposed to 0, 0.1, 1, and 6 mg/kg body weight/day of the MARBLES mix shows not separation by exposure group. Veh, vehicle.

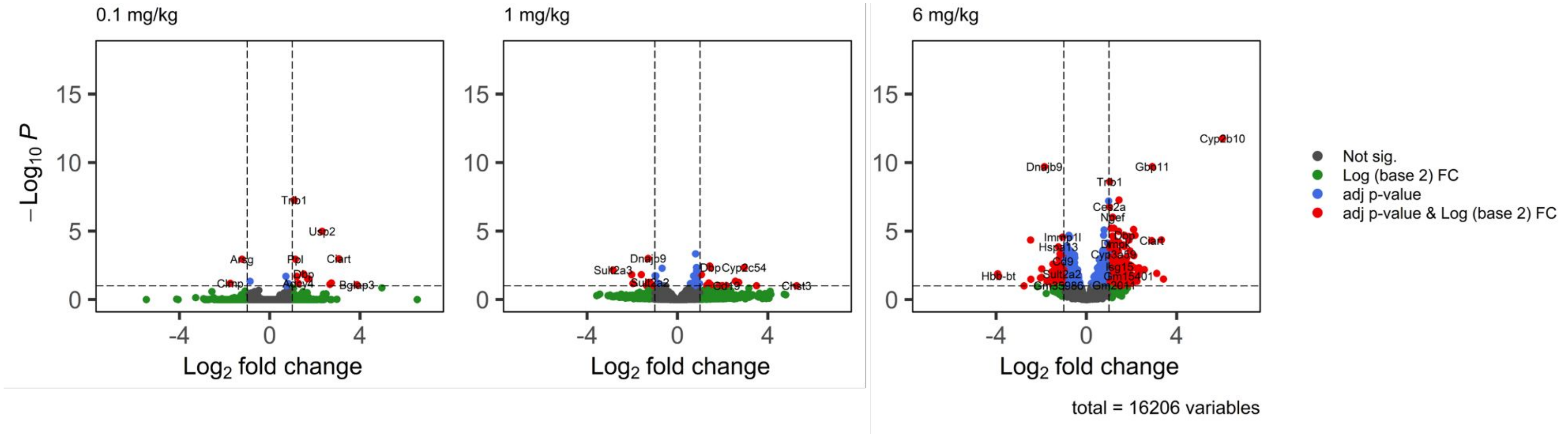

Fig S6. Volcano plots showing pairwise comparisons of RNA sequencing data from each MARBLES mix dose group versus the vehicle group in the liver. Thresholds adjusted p-value<0.1 and $\log_2$ fold change >1. Plots were generated using the *EnhancedVolcano* package.

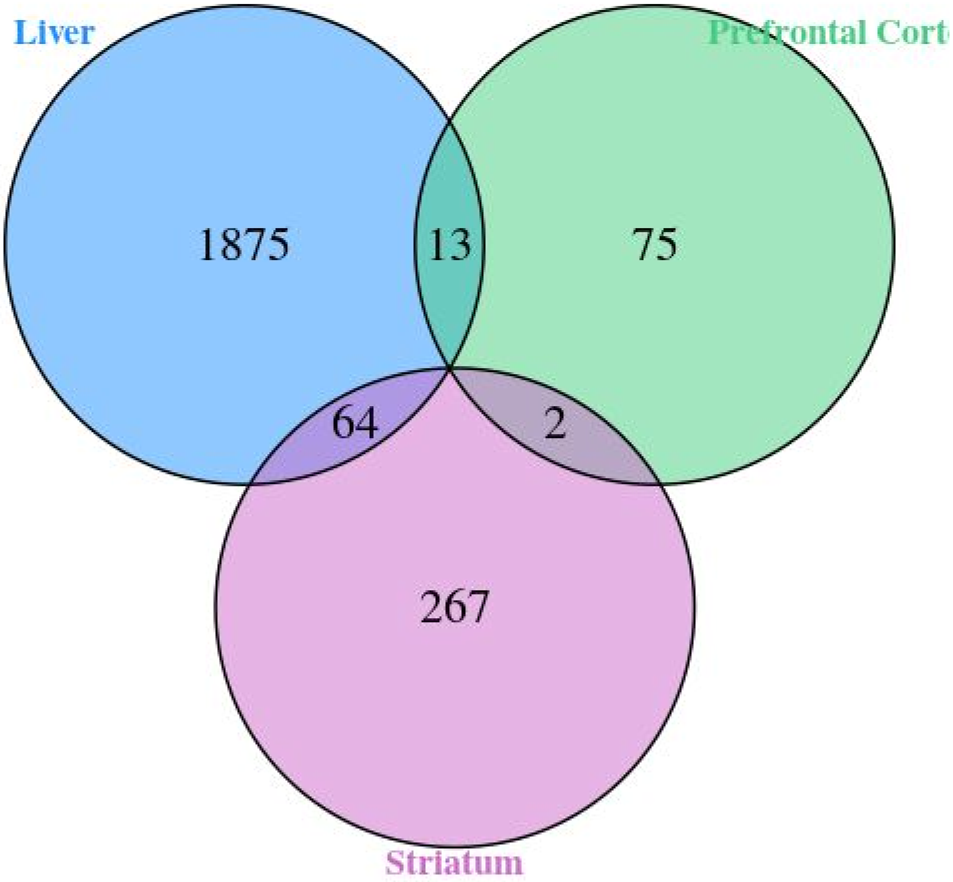

Fig S7. Venn diagram of common differentially expressed genes across tissue samples collected from mice exposed to 6 mg/kg of the MARBLES mix. The Venn diagram was generated using the *VennDetail* shiny app, using an adjusted p-values <0.1 as a threshold.

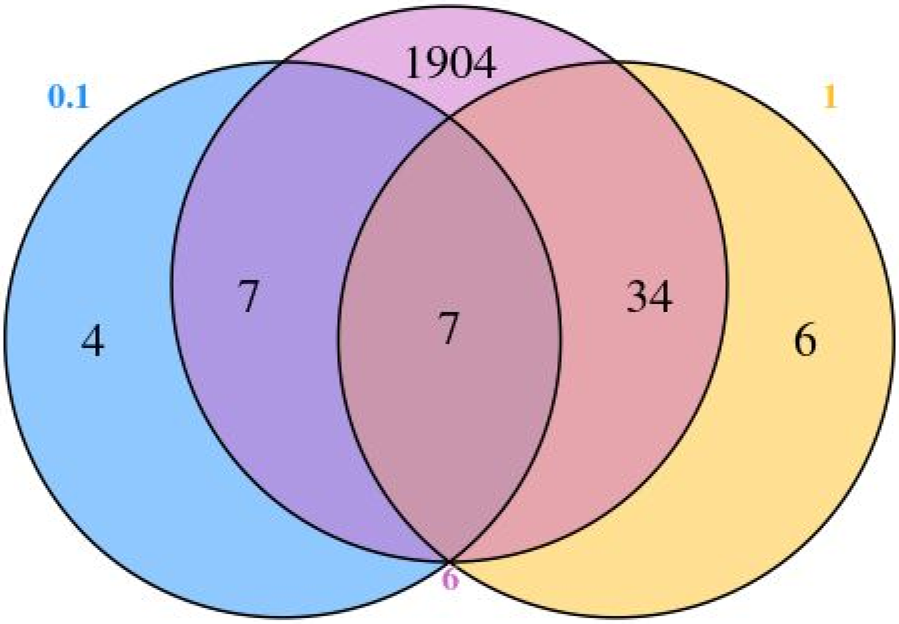

Fig S8. Venn diagram of common differentially expressed genes across exposure groups in the liver samples collected from mice exposed to 0.1, 1, and 6 mg/kg body weight/day of the MARBLES mix. For normalized gene count plots of the seven differentially expressed genes in all three PCB doses, see **Fig S9**. The Venn diagram was generated using the *VennDetail* shiny app, using an adjusted p-values <0.1 as a threshold.

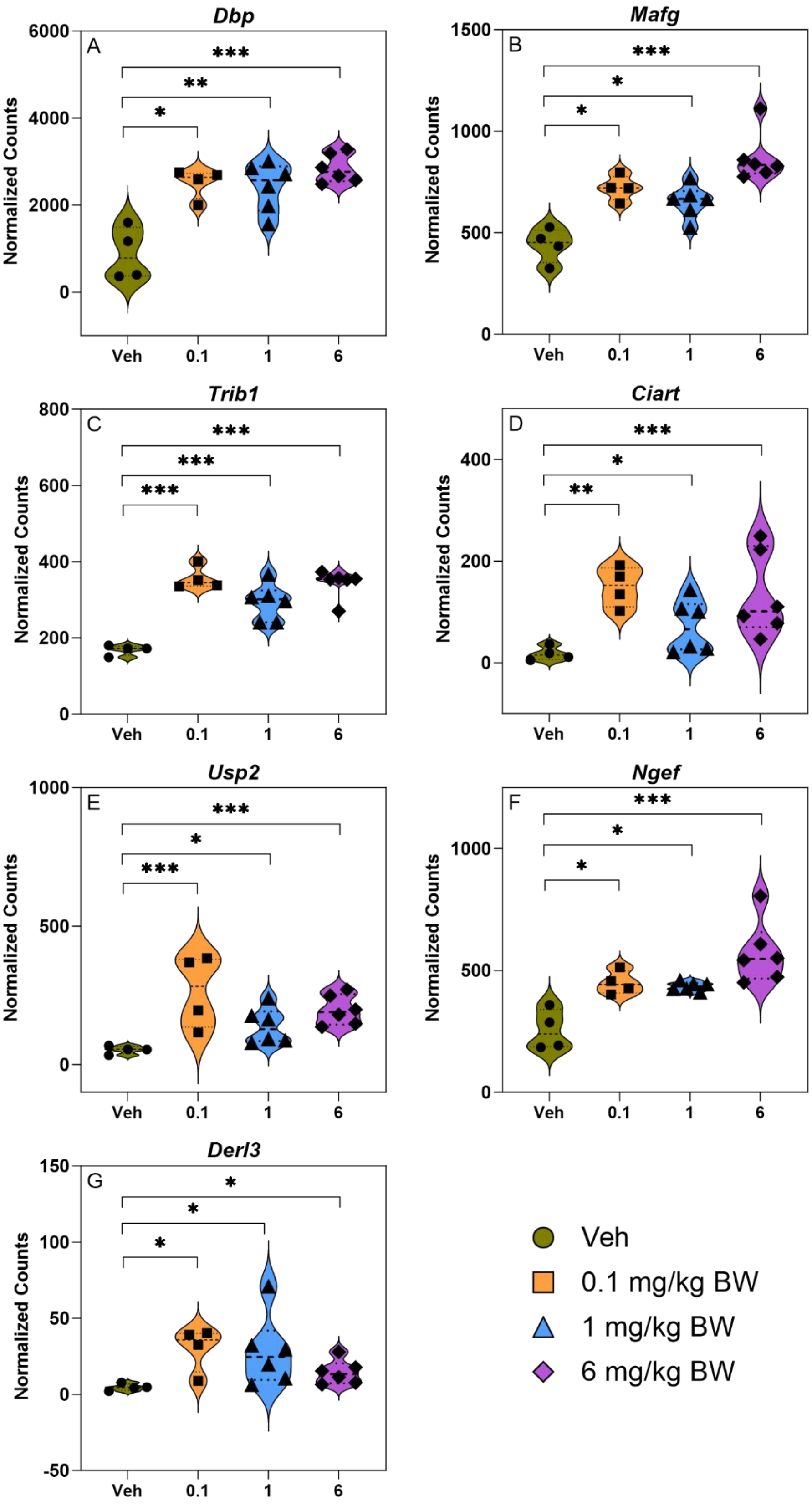

Dbp
Mafg
Trib1
Ciart
Usp2
Ngef
Derl3
A
B
C
D
E
F
G
Normalized Counts
Veh
0.1
1
6
Veh
0.1 mg/kg BW
1 mg/kg BW
6 mg/kg BW

Fig S9. Normalized gene count plots of the seven differentially expressed genes identified in the Venn diagram in **Fig S8**, including (A) D-site albumin promoter binding protein (*Dbp*), (B) MAF BZIP transcription factor G (*Mafg*), (C) Tribbles pseudokinase 1 (*Trib1*), (D) circadian associated repressor of transcription (*Ciart*), (E) ubiquitin specific peptidase (*Usp2*), (F) neuronal guanine nucleotide exchange factor (*Ngef*), and (G) derlin 3 (*Derl3*). *Dbp* and *Ciart* are auxiliary components regulating the circadian clock.[24] Several studies have reported alterations in the circadian rhythm following exposure to PCBs and their metabolites in zebrafish embryos or mice.[25, 26] Moreover, *Usp2* and *Derl3* are associated with ubiquitin pathways[27] that regulate core clock components and other clock protein functions.[28] These observations suggest that PCB exposure altered the circadian clock in the liver. Violin plots are expressed as mean ± standard error with individual dots representing different animals. (N = 4 to 6). For a description of the statistical analysis, see the manuscript. * adjusted p-value <0.1, ** adjusted p-value <0.01, *** adjusted p-value <0.001.

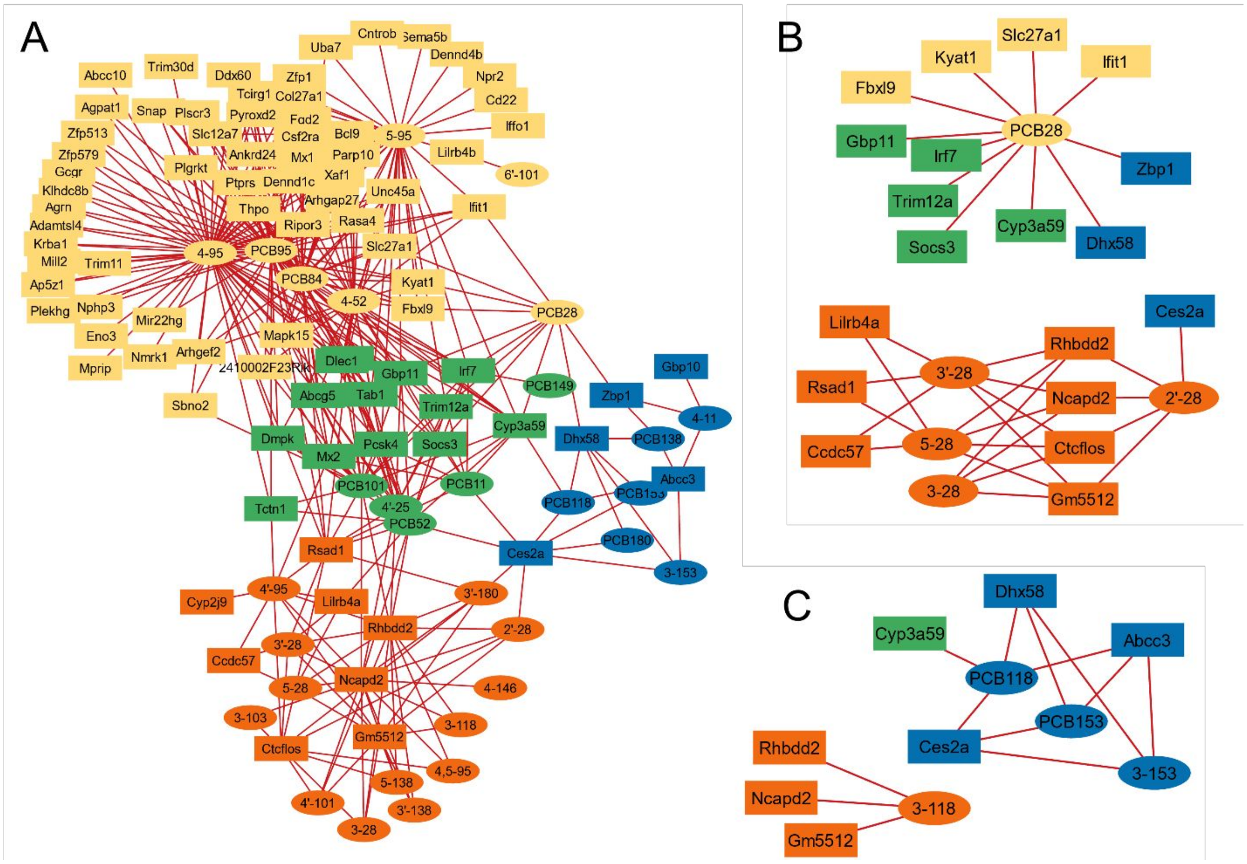

Fig S10. Network analysis of liver transcriptome and liver PCB and OH-PCB levels identifying four clusters (**panel A**). Eighty-four genetic transcripts and 31 PCBs had strong and significant correlations in the network ($|r| > 0.85$, $p < 0.05$). Notably, all identified genes were positively correlated to PCBs at this threshold. The gene that showed a strong link with most PCBs is non-SMC condensin I complex subunit D2 (*Ncapd2*). *Ncapd2* plays a role in promoting cancer progression and leading to poor prognosis.[29] **Panel B** depicts a sub-network of genes correlated with PCB28 and OH-metabolites. Surprisingly, PCB28 and its OH-metabolites have no shared genes, resulting in complete separation in the networks. **Panel C** illustrates the genes correlated with PCB118, PCB153, and their hydroxyl metabolites. ATP-binding cassette sub-family C member 3 (Abcc3), also known as multidrug resistance-associated protein 3 (MRP3), positively interconnects with PCB118 and PCB153. Both PCB congeners are structurally similar, with

PCB153 having one additional *ortho* chlorine substituent. Abcc3 is a transporter protein involved in the efflux of various compounds from cells. It may play a role in the cellular detoxification of PCBs, especially those conjugated with UDP-glucuronic acid or glutathione.[30] Carboxylesterase 2a (*Ces2a*), another gene positively interconnected with PCB118 and PCB153, plays a significant role in xenobiotic metabolism. A study also reported the dose-dependent induction of *Abcc3* and *Ces2* after PCB153 was administered by gavage in immature, ovariectomized C57BL/6 mice.[31] Further studies are needed to determine if PCB118 and PCB153 induce the expression of transporters involved in eliminating PCB metabolites from the liver. Network analyses were performed with xMWAS (version 0.552)[32] using a threshold of absolute correlation coefficients > 0.85 and $P < 0.05$. Nodes in the same cluster share the same color. The node shape represents PCBs (ovals) and genes (rectangles). The edge color indicates positive (red) correlations.

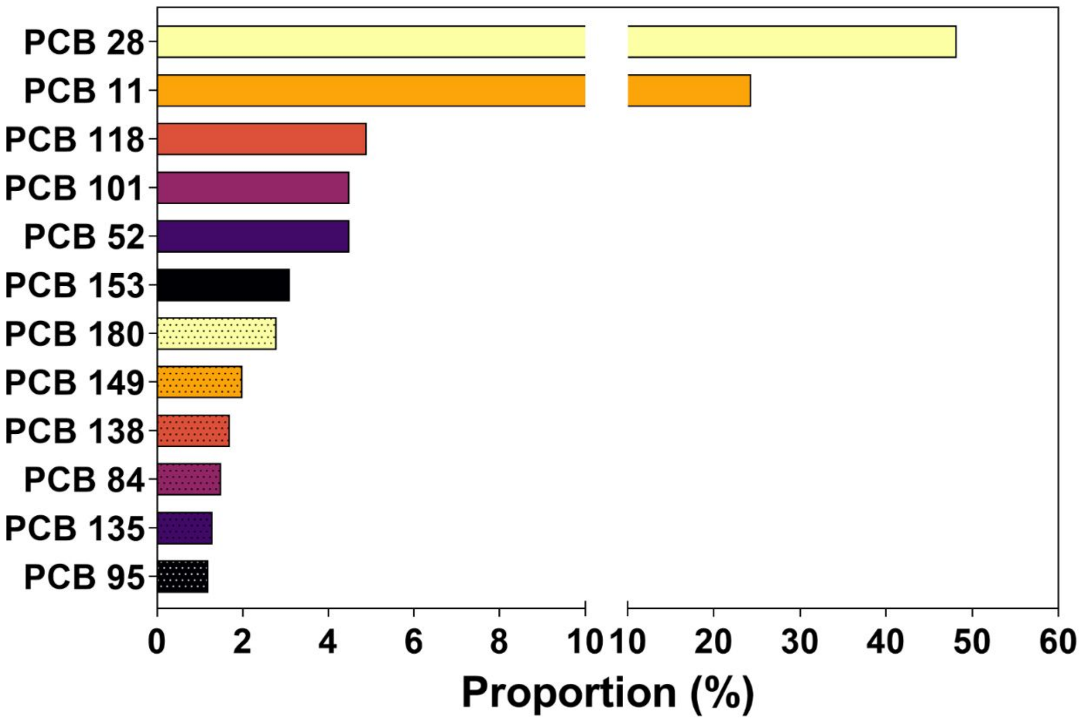

Fig S11. Mass profile of the MARBLES mix, a PCB mixture containing 12 PCB congeners detected in serum collected during the third trimester of women enrolled in the MARBLES study.

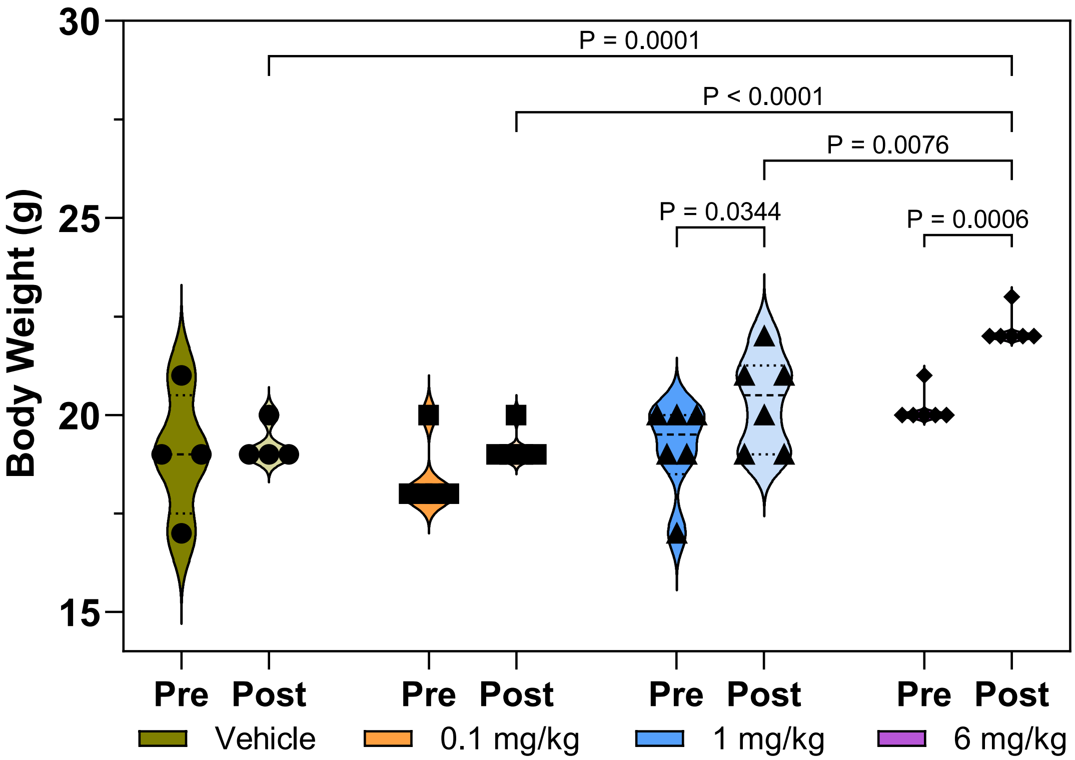

Fig S12. Pre- and post-exposure body weights of female mice exposed to different doses of the MARBLES mix. Violin plots show group variability with color and shape corresponding to the exposure group. Pre- and post-weight differences between exposure groups were assessed by two-way ANOVA followed by Tukey post hoc for multiple comparisons.

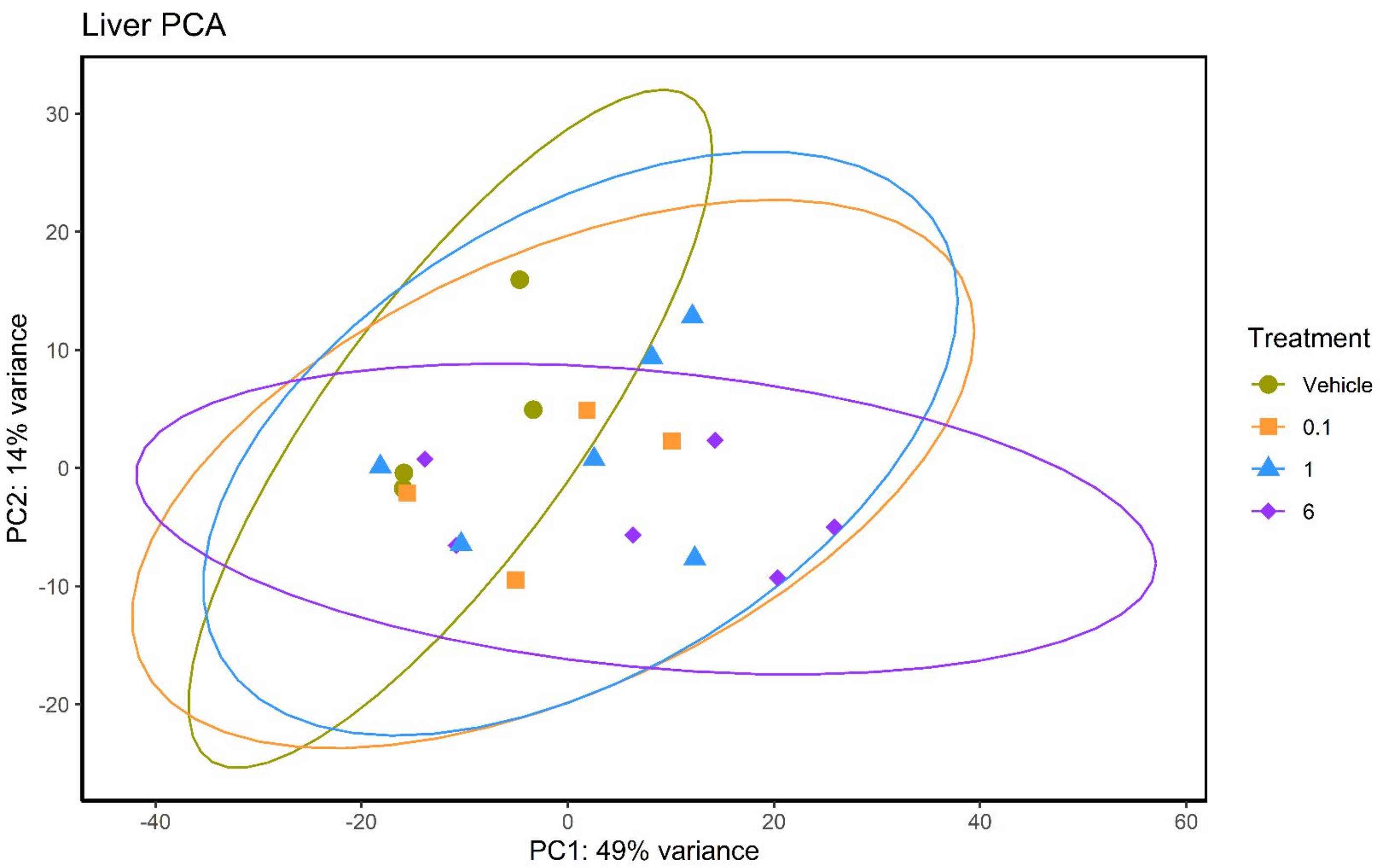

Fig S13. Principal component analysis (PCA) plot of RNA sequencing data from liver samples from mice exposed to 0, 0.1, 1, or 6 mg/kg body weight/day of the MARBLES mix. The first two principal components derived from the analysis to explain the sample variance are plotted. The exposure groups are identified by different shapes and colors.

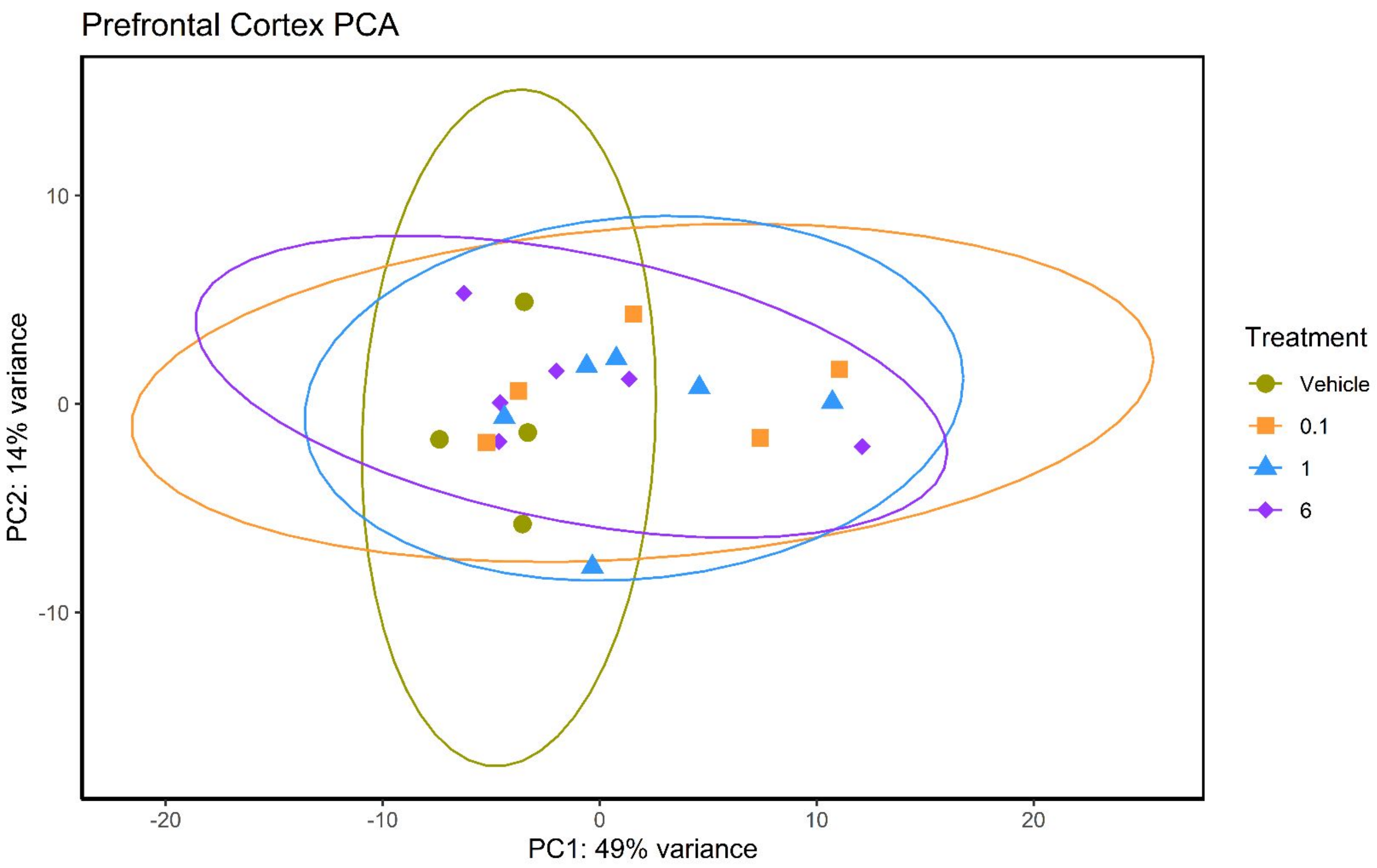

Fig S14. Principal component analysis (PCA) plot of RNA sequencing data from prefrontal cortex samples from mice exposed to 0, 0.1, 1, or 6 mg/kg body weight/day of the MARBLES mix. The first two principal components derived from the analysis to explain the sample variance are plotted. The exposure groups are identified by different shapes and colors.

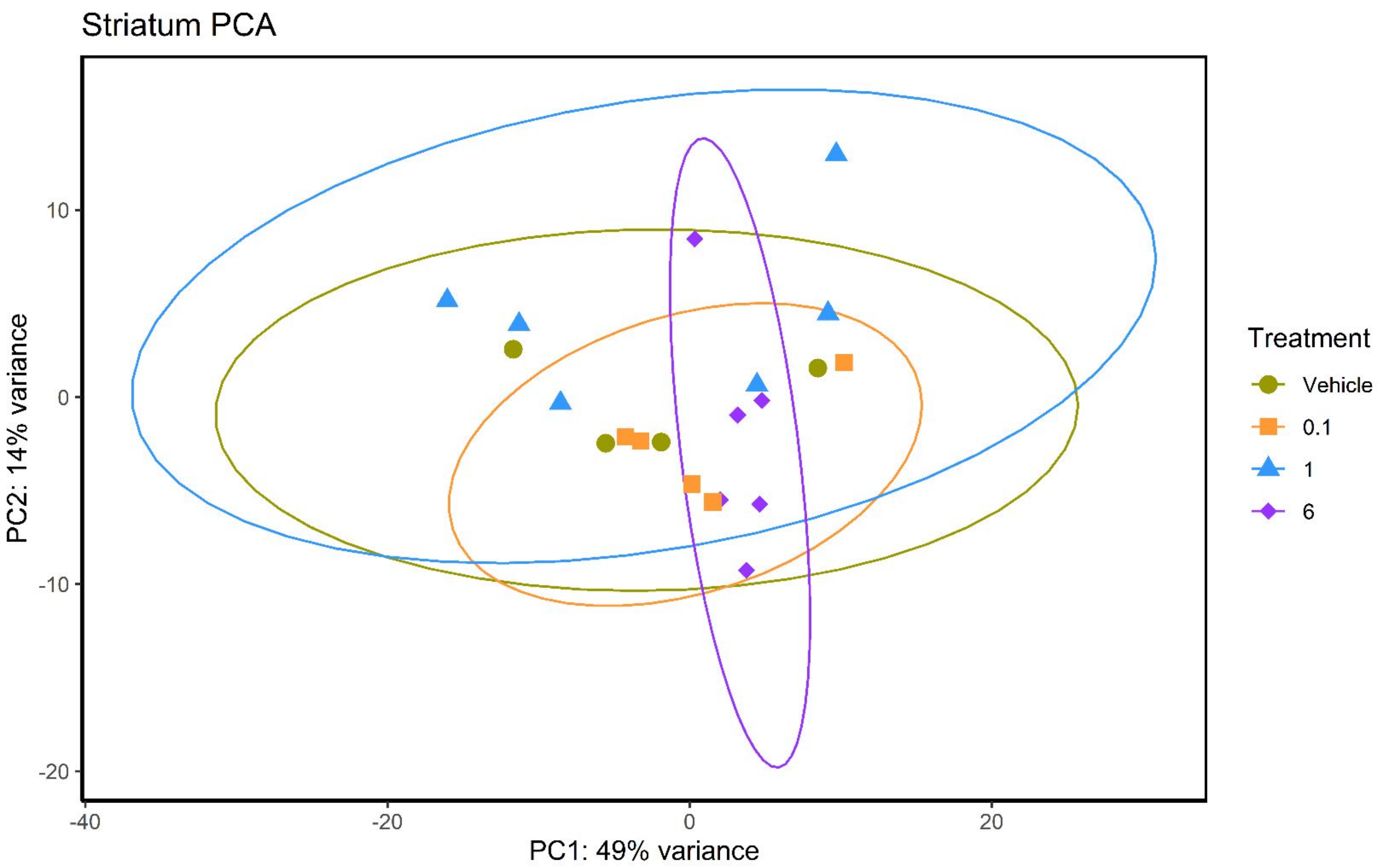

Fig S15. Principal component analysis (PCA) plot of RNA sequencing data from striatum samples from mice exposed to 0, 0.1, 1, or 6 mg/kg body weight/day of the MARBLES mix. The first two principal components derived from the analysis to explain the sample variance are plotted. The exposure groups are identified by different shapes and colors.

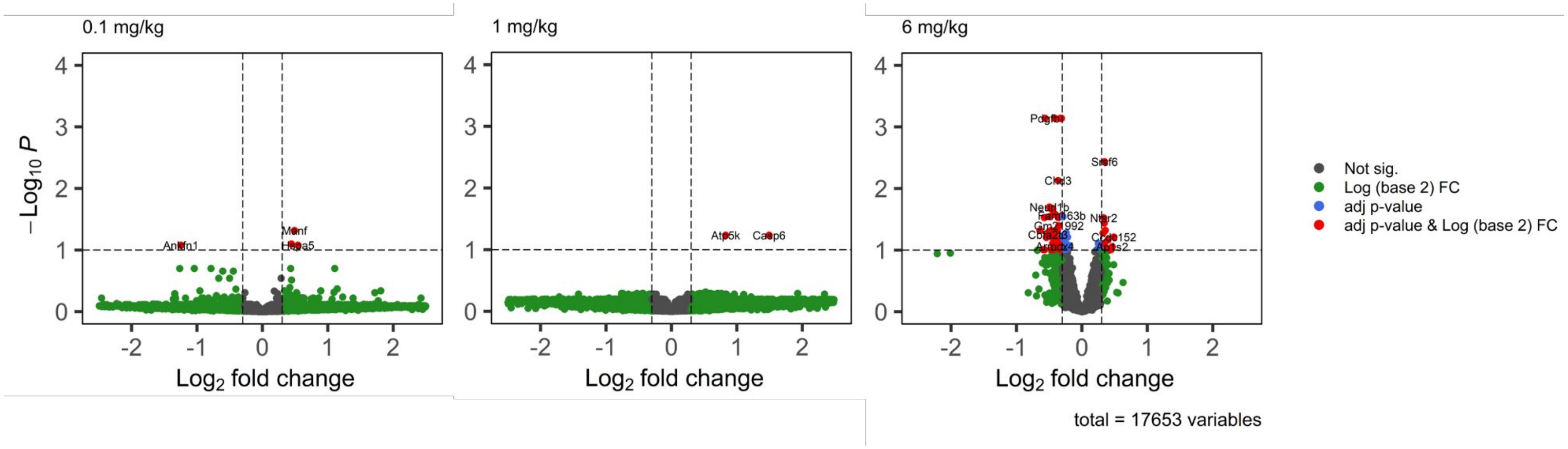

Fig S16. Volcano plots showing pairwise comparisons of RNA sequencing data from each MARBLES mix dose group versus the vehicle group in the prefrontal cortex. Thresholds adjusted p-value<0.1 and $\log_2$ fold change >0.3. Plots generated using *EnhancedVolcano* package.

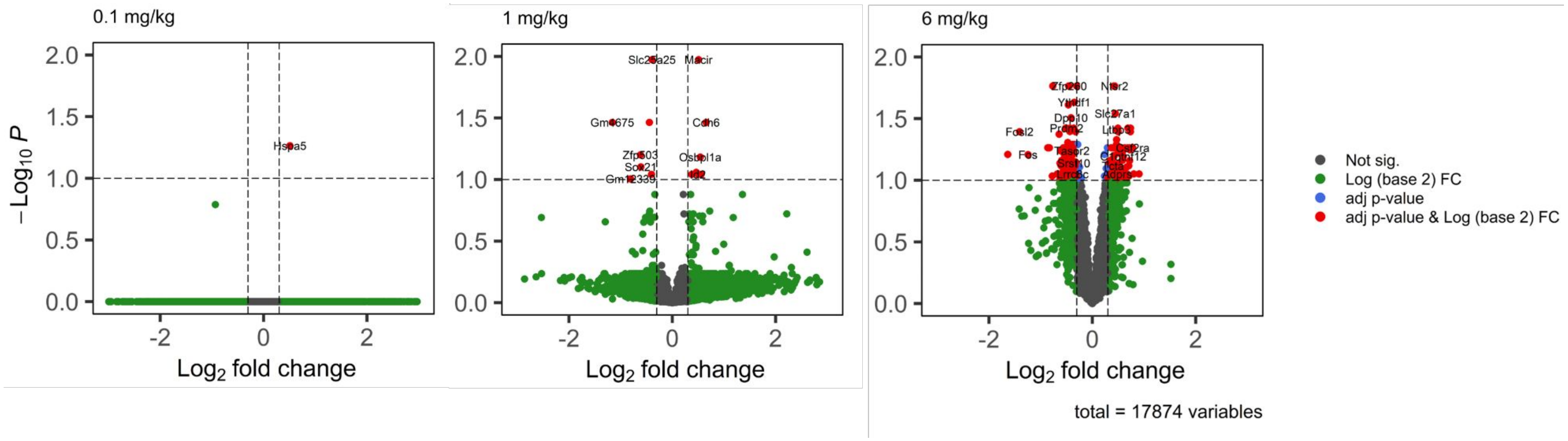

Fig S17. Volcano plots showing pairwise comparisons of RNA sequencing data from each MARBLES mix dose group versus the vehicle group in the striatum. Thresholds adjusted p-value<0.1 and $\log_2$ fold change >0.3. Plots generated using *EnhancedVolcano* package.

References

1. US EPA, Table of Polychlorinated Biphenyl (PCB) Congeners. https://www.epa.gov/pcbs/table-polychlorinated-biphenyl-pcb-congeners (accessed July 27, 2024),
2. Maervoet, J.; Covaci, A.; Schepens, P.; Sandau, C. D.; Letcher, R. J., A reassessment of the nomenclature of polychlorinated biphenyl (PCB) metabolites. *Environ Health* Perspect 2004, 112, (3), 291-4.
3. Li, X.; Behan-Bush, R.M.; Liszewski, J.N.; Schrodt, M.V.; Vats, B.; Klingelhutz, A.J.; Ankrum, J.A.; Lehmler, H.-J., The preparation and authentication of Cabinet Mixture. University of Iowa (dataset), 2022. https://doi.org/10.25820/data.006184.
4. Zhang, C. Y.; Li, X.; Keil Stietz, K. P.; Sethi, S.; Yang, W.; Marek, R. F.; Ding, X.; Lein, P. J.; Hornbuckle, K. C.; Lehmler, H. J., Machine learning-assisted identification and quantification of hydroxylated metabolites of polychlorinated biphenyls in animal samples. Environ Sci Technol 2022, 56, (18), 13169-13178.
5. Marek, R. F.; Thorne, P. S.; Herkert, N. J.; Awad, A. M.; Hornbuckle, K. C., Airborne PCBs and OH-PCBs inside and outside urban and rural U.S. schools. *Environ Sci Technol* 2017, *51*, (14), 7853-7860.
6. Lehmler, H. J.; Robertson, L. W., Synthesis of polychlorinated biphenyls (PCBs) using the Suzuki-coupling. *Chemosphere* 2001, *45*, (2), 137-143.
7. Lehmler, H. J.; Robertson, L. W., Synthesis of hydroxylated PCB metabolites with the Suzuki-coupling. *Chemosphere* 2001, *45*, (8), 1119-1127.
8. Dhakal, R.; Li, X.; Parkin, S. R.; Lehmler, H.-J., Synthesis of mono-and dimethoxylated polychlorinated biphenyls derivatives starting from fluoroarene derivatives. *Environ Sci Pollut Res* 2020, *27*, 8905-8925.
9. Li, X.; Holland, E. B.; Feng, W.; Zheng, J.; Dong, Y.; Pessah, I. N.; Duffel, M. W.; Robertson, L. W.; Lehmler, H. J., Authentication of synthetic environmental contaminants and their (bio)transformation products in toxicology: polychlorinated biphenyls as an example. *Environ Sci Pollut Res* 2018, *25*, (17), 16508-16521.
10. McLean, M. R.; Bauer, U.; Amaro, A. R.; Robertson, L. W., Identification of catechol and hydroquinone metabolites of 4-monochlorobiphenyl. *Chem Res Toxicol* 1996, *9*, (1), 158-164.
11. Rodriguez, E. A.; Li, X.; Lehmler, H. J.; Robertson, L. W.; Duffel, M. W., Sulfation of lower chlorinated polychlorinated biphenyls increases their affinity for the major drug-binding sites of human serum albumin. *Environ Sci Technol* 2016, *50*, (10), 5320-5327.
12. Saktrakulkla, P.; Dhakal, R. C.; Lehmler, H. J.; Hornbuckle, K. C., A semi-target analytical method for quantification of OH-PCBs in environmental samples. *Environ Sci Pollut Res* 2020, *27*, (9), 8859-8871.
13. Zhai, G. S.; Lehmler, H. J.; Schnoor, J. L., New hydroxylated metabolites of 4-monochlorobiphenyl in whole poplar plants. *Chem Cent J* 2011, *5*.
14. Zhu, Y. M.; Mapuskar, K. A.; Marek, R. F.; Xu, W. J.; Lehmler, H. J.; Robertson, L. W.; Hornbuckle, K. C.; Spitz, D. R.; Aykin-Burns, N., A New Player in Environmentally Induced Oxidative Stress: Polychlorinated Biphenyl Congener, 3,3-Dichlorobiphenyl (PCB11). *Toxicol Sci* 2013, *136*, (1), 39-50.
15. Alam, S.; Carter, G.; Krager, K.; Li, X.; Lehmler, H.-J.; Aykin-Burns, N., PCB11 metabolite, 3,3'-dichlorobiphenyl-4-ol, exposure alters the expression of genes governing

fatty acid metabolism in the absence of functional sirtuin 3: examining the contribution of MnSOD. *Antioxidants* 2018, *7*, (9), E121.

16. Joshi, S. N.; Vyas, S. M.; Duffel, M. W.; Parkin, S.; Lehmler, H.-J., Synthesis of sterically hindered polychlorinated biphenyl derivatives. *Synthesis* 2011, (7), 1045-1054.
17. Li, X.; Parkin, S.; Duffel, M. W.; Robertson, L. W.; Lehmler, H.-J., An efficient approach to sulfate metabolites of polychlorinated biphenyls. *Environ Int* 2010, *36*, 843-848.
18. Black, T. H., The preparation and reactions of diazomethane. *Aldrichim Acta* 1983, *16*, (1), 3-10.
19. Love, M. I.; Huber, W.; Anders, S., Moderated estimation of fold change and dispersion for RNA-seq data with DESeq2. *Genome Biol* 2014, *15*, (12), 550.
20. Nguyen, T. M.; Shafi, A.; Nguyen, T.; Draghici, S., Identifying significantly impacted pathways: a comprehensive review and assessment. *Genome Biol* 2019, *20*, (1), 203.
21. Maleki, F.; Ovens, K.; Hogan, D. J.; Kusalik, A. J., Gene set analysis: challenges, opportunities, and future research. *Front Genet* 2020, *11*, 654.
22. Wu, T.; Hu, E.; Xu, S.; Chen, M.; Guo, P.; Dai, Z.; Feng, T.; Zhou, L.; Tang, W.; Zhan, L.; Fu, X.; Liu, S.; Bo, X.; Yu, G., clusterProfiler 4.0: A universal enrichment tool for interpreting omics data. *Innovation (Camb)* 2021, *2*, (3), 100141.
23. Wang, X.; Park, J.; Susztak, K.; Zhang, N. R.; Li, M., Bulk tissue cell type deconvolution with multi-subject single-cell expression reference. *Nat Commun* 2019, *10*, (1), 380.
24. Sato, S.; Bunney, B.; Mendoza-Viveros, L.; Bunney, W.; Borrelli, E.; Sassone-Corsi, P.; Orozco-Solis, R., Rapid-acting antidepressants and the circadian clock. *Neuropsychopharmacology* 2022, *47*, (4), 805-816.
25. Aluru, N.; Karchner, S. I.; Glazer, L., Early life exposure to low levels of AHR agonist PCB126 (3,3',4,4',5-pentachlorobiphenyl) reprograms gene expression in adult brain. *Toxicol Sci* 2017, *160*, (2), 386-397.
26. Shen, X.; Chen, Y.; Zhang, J.; Yan, X.; Liu, W.; Guo, Y.; Shan, Q.; Liu, S., Low-dose PCB126 compromises circadian rhythms associated with disordered glucose and lipid metabolism in mice. *Environ Int* 2019, *128*, 146-157.
27. Abdalla, O.; Mascarenhas, B.; Cheng, H. M., Death of a protein: the role of E3 ubiquitin ligases in circadian rhythms of mice and flies. *Int J Mol Sci* 2022, *23*, (18).
28. Stojkovic, K.; Wing, S. S.; Cermakian, N., A central role for ubiquitination within a circadian clock protein modification code. *Front Mol Neurosci* 2014, *7*.
29. Zhu, S.; Qiu, H.; Bennett, S.; Kuek, V.; Rosen, V.; Xu, H.; Xu, J., Chondromodulin-1 in health, osteoarthritis, cancer, and heart disease. *Cell Mol Life Sci* 2019, *76*, (22), 4493-4502.
30. Epel, D.; Luckenbach, T.; Stevenson, C. N.; Macmanus-Spencer, L. A.; Hamdoun, A.; Smital, T., Efflux transporters: newly appreciated roles in protection against pollutants. *Environ Sci Technol* 2008, *42*, (11), 3914-20.
31. Kopec, A. K.; Burgoon, L. D.; Ibrahim-Aibo, D.; Mets, B. D.; Tashiro, C.; Potter, D.; Sharratt, B.; Harkema, J. R.; Zacharewski, T. R., PCB153-elicited hepatic responses in the immature, ovariectomized C57BL/6 mice: comparative toxicogenomic effects of dioxin and non-dioxin-like ligands. *Toxicol Appl Pharmacol* 2010, *243*, (3), 359-71.
32. Uppal, K.; Ma, C.; Go, Y. M.; Jones, D. P.; Wren, J., xMWAS: a data-driven integration and differential network analysis tool. *Bioinformatics* 2018, *34*, (4), 701-702.
